# Control of vein-forming, striped gene expression by auxin signaling

**DOI:** 10.1186/s12915-021-01143-9

**Published:** 2021-09-24

**Authors:** Anmol Krishna, Jason Gardiner, Tyler J. Donner, Enrico Scarpella

**Affiliations:** 1 Department of Biological Sciences, University of Alberta, CW-405 Biological Sciences Building, Edmonton, AB T6G 2E9 Canada; 2 Present Address — Department of Molecular, Cell, and Developmental Biology, University of California, Los Angeles, CA 90095 USA; 3 Present Address — Department of Biology, University of British Columbia, Kelowna, BC V1V 1V7 Canada

**Keywords:** Stripe formation, Gene regulatory network, *Arabidopsis thaliana*, Auxin, Leaf vascular patterning, *MONOPTEROS*, *ARABIDOPSIS THALIANA HOMEOBOX8*, Incoherent feedforward loop, Vein network formation

## Abstract

**Background:**

Activation of gene expression in striped domains is a key building block of biological patterning, from the recursive formation of veins in plant leaves to that of ribs and vertebrae in our bodies. In animals, gene expression is activated in striped domains by the differential affinity of broadly expressed transcription factors for their target genes and the combinatorial interaction between such target genes. In plants, how gene expression is activated in striped domains is instead unknown. We address this question for the broadly expressed MONOPTEROS (MP) transcription factor and its target gene *ARABIDOPSIS THALIANA HOMEOBOX FACTOR8* (*ATHB8*).

**Results:**

We find that *ATHB8* promotes vein formation and that such vein-forming function depends on both levels of *ATHB8* expression and width of *ATHB8* expression domains. We further find that *ATHB8* expression is activated in striped domains by a combination of (1) activation of *ATHB8* expression through binding of peak levels of MP to a low-affinity MP-binding site in the *ATHB8* promoter and (2) repression of *ATHB8* expression by MP target genes of the *AUXIN*/*INDOLE-3-ACETIC-ACID-INDUCIBLE* family.

**Conclusions:**

Our findings suggest that a common regulatory logic controls activation of gene expression in striped domains in both plants and animals despite the independent evolution of their multicellularity.

**Supplementary Information:**

The online version contains supplementary material available at 10.1186/s12915-021-01143-9.

## Background

Narrow stripes of gene expression are fundamental units of biological patterning (e.g., [1–3]). Therefore, how multicellular organisms activate gene expression in narrow stripes is a central question in biology. In animals, where this question has been investigated extensively, broadly expressed transcription factors activate expression of their target genes in narrow stripes by (1) differential affinity of such transcription factors for their binding sites in target genes and (2) combinatorial interactions between transcription-factor-encoding target genes [4–7]. For example, the transcription factor Dorsal forms a ventral-to-dorsal gradient in Drosophila embryos (reviewed in [8]). Expression of Dorsal target genes with high-affinity Dorsal-binding sites is activated already at low levels of Dorsal, whereas expression of Dorsal target genes with low-affinity Dorsal-binding sites is activated only at high levels of Dorsal. However, this mechanism alone is insufficient to account for the expression of Dorsal target genes in stripes: interaction between Dorsal target genes themselves is also required: Dorsal activates expression of *snail*, which encodes a transcription factor that represses the expression of the Dorsal target gene *ventral nervous system defective*. Thus, expression of some Dorsal target genes such as *ventral nervous system defective* is repressed at high levels of Dorsal, at which *snail* is expressed, but activated at lower levels of Dorsal, at which *snail* is not expressed.

In plants too, broadly expressed transcription factors activate expression of their target genes in narrow stripes (e.g., [9]); however, how these broadly expressed transcription factors do so is unclear. Here we addressed this question for the *MONOPTEROS* (*MP*) – *ARABIDOPSIS THALIANA HOMEOBOX8* (*ATHB8*) pair of Arabidopsis genes [10, 11]. *ATHB8* expression is activated in single files of isodiametric ground cells of the leaf [12, 13]. *ATHB8*-expressing ground cells will elongate into procambial cells — the precursors to all vascular cells — and are therefore referred to as preprocambial cells [12–15]. Activation of *ATHB8* expression in narrow preprocambial stripes depends on binding of the broadly expressed MP transcription factor to a low-affinity MP-binding site in the *ATHB8* promoter [16]. However, the biological relevance of activation of *ATHB8* expression by MP is unclear: whereas *MP* promotes vein formation [17], *ATHB8* seems to have only transient and conditional functions in vein network formation [16, 18].

Here we show that *ATHB8* promotes vein formation and that both levels of *ATHB8* expression and width of *ATHB8* expression domains are relevant to vein formation. Finally, we show that *ATHB8* expression is restricted to narrow preprocambial stripes by a combination of (1) activation of *ATHB8* expression through binding of peak levels of MP to a low-affinity MP-binding site in the *ATHB8* promoter and (2) repression of *ATHB8* expression by MP target genes of the *AUXIN*/*INDOLE-3-ACETIC-ACID-INDUCIBLE* family.

## Results

### Response of vein network formation to changes in ATHB8 expression and activity

To understand how in plants broadly expressed transcription factors activate expression of their target genes in narrow stripes, we chose the *MP* – *ATHB8* pair of Arabidopsis genes. During leaf development, the broadly expressed MP transcription factor directly activates *ATHB8* expression in narrow preprocambial stripes that mark the position where veins will form [16], but the biological relevance of the interaction between the two genes is unclear.

That *MP* promotes vein formation is known [17], but the function of *ATHB8* in this process is unresolved: *athb8* mutants seem to have only transient and conditional defects in vein network formation, and the mutants have normal vein patterns [16, 18]. Therefore, we first asked whether *ATHB8* had any permanent functions in vein network formation. To address this question, we characterized the vein networks in mature first leaves of the *athb8-11* and *athb8-27* loss-of-function mutants [19] (Table S1) — and of other genotypes in our study — by means of four descriptors: a cardinality index, a continuity index, and a connectivity index [20], and a cyclicity index.

The cardinality index is a proxy for the number of “veins” (i.e., stretches of vascular elements that contact other stretches of vascular elements at least at one of their two ends) in a network. The continuity index quantifies how close a vein network is to a network with the same pattern but in which at least one end of each “vein fragment” (i.e., a stretch of vascular elements that is free of contact with other stretches of vascular elements) contacts a vein. The connectivity index quantifies how close a vein network is to a network with the same pattern but in which both ends of each vein or vein fragment contact other veins. The cyclicity index is a proxy for the number of meshes in a vein network.

**Fig. 1:**
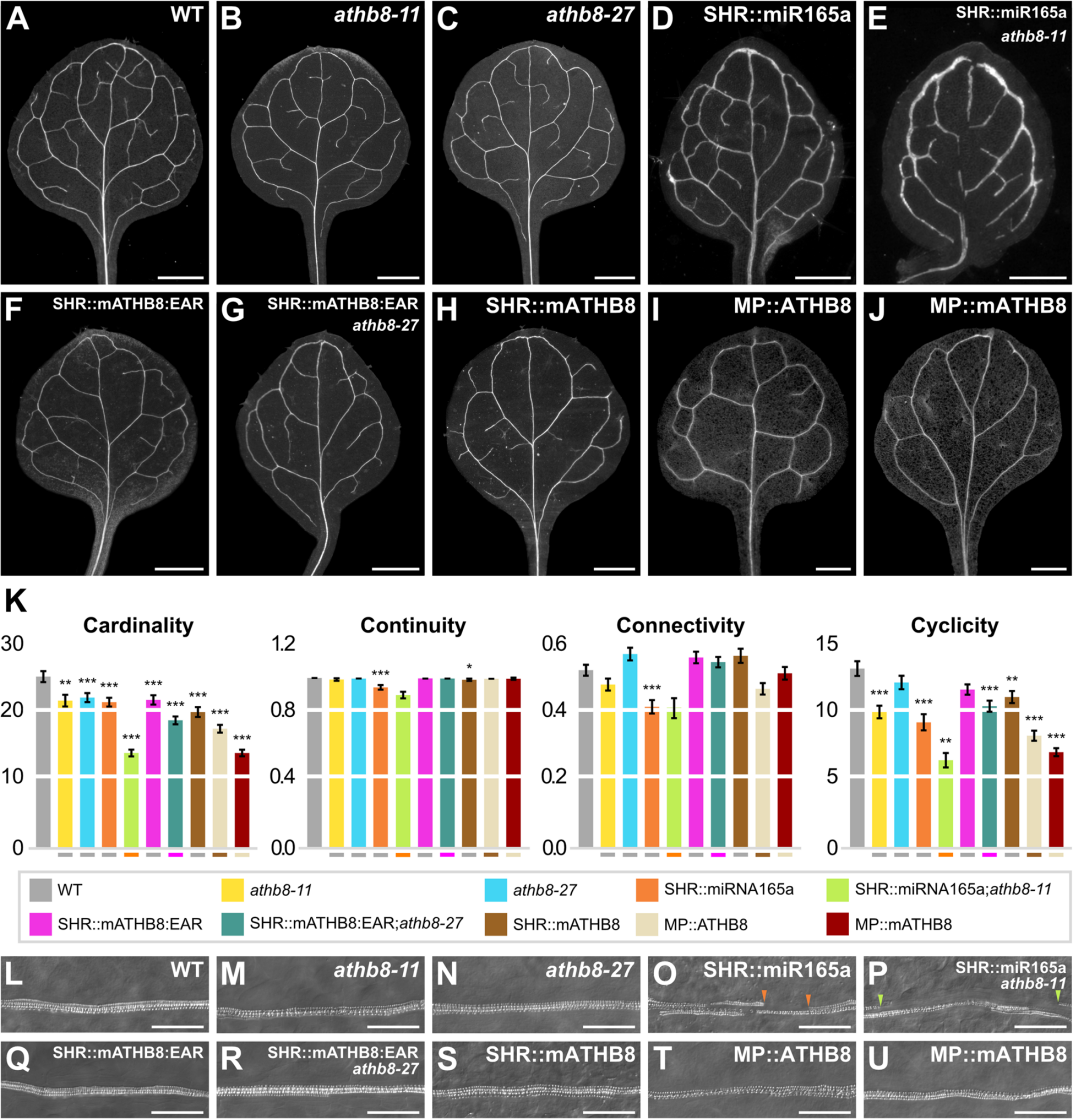
*ATHB8* Function in Vein Network Formation. (**A**–**J**,**L**–**U**) Dark-field (**A**–**J**) or Differential-interfering-contrast (**L**–**U**) illumination of cleared first leaves 14 days after germination (DAG). Top right: genotype. (**K**) Cardinality, connectivity, and continuity index (mean ± SE) as defined in [20] and Methods; cyclicity index (mean ± SE) as defined in Methods. Test genotypes were compared with the reference genotypes represented by the lines under the bars. Each index in *athb8-11*, *athb8-27*, SHR::miR165a, SHR::mATHB8:EAR, and SHR::mATHB8 was compared with the respective index in WT. Each index in SHR::miR165a;*athb8-11* was compared with the respective index in SHR::miR165a. Each index in SHR::mATHB8:EAR;*athb8-27* was compared with the respective index in SHR::mATHB8:EAR. Each index in MP::ATHB8 was compared with the respective index in SHR::mATHB8. Each index in MP::mATHB8 was compared with the respective index in MP::ATHB8. Difference between *athb8-11* and WT cardinality indices, between *athb8-27* and WT cardinality indices, between SHR::miR165a and WT cardinality indices, between SHR::miR165a;*athb8-11* and SHR::miR165a cardinality indices, between SHR::mATHB8:EAR and WT cardinality indices, between SHR::mATHB8:EAR;*athb8-27* and SHR::mATHB8:EAR cardinality indices, between SHR::mATHB8 and WT cardinality indices, between MP::ATHB8 and SHR::mATHB8 cardinality indices, between MP::mATHB8 and MP::ATHB8 cardinality indices, between SHR::miR165a and WT continuity indices, between SHR::mATHB8 and WT continuity indices, between SHR::miR165a and WT connectivity indices, between *athb8-11* and WT cyclicity indices, between SHR::miR165a and WT cyclicity indices, between SHR::miR165a;*athb8-11* and SHR::miR165a cyclicity indices, between SHR::mATHB8:EAR;*athb8-27* and SHR::mATHB8:EAR cyclicity indices, between SHR::mATHB8 and WT cyclicity indices, between MP::ATHB8 and SHR::mATHB8 cyclicity indices, and between MP::mATHB8 and MP::ATHB8 cyclicity indices was significant at *P* *<* 0.05 (*), *P* *<* 0.01 (**), or *P* *<* 0.001 (***) by *F*-test and *t*-test with Bonferroni correction. Sample sizes: WT, 58; *athb8-11*, 39; *athb8-27*, 32; SHR::miR165a, 51; SHR::miR165a;*athb8-11*, 64; SHR::mATHB8:EAR, 38; SHR::mATHB8:EAR;*athb8-27*, 28; SHR::mATHB8, 33; MP::ATHB8, 37; MP::mATHB8, 47. (**L**–**U**) Details of the upper fourth of the midvein. Arrowheads indicate gaps in xylem differentiation. Scale bars: (**A**,**I**,**J**) 0.5 mm; (**B**,**C**,**F**,**G**,**H**) 1 mm; (**D**,**E**) 0.2 mm; (**L**–**U**) 50 μm

The cardinality index of both *athb8-11* and *athb8-27* was lower than that of wild type (WT) (Fig. 1A–C,K), suggesting that *ATHB8* promotes vein formation.

*ATHB8* encodes a transcription factor member of the HOMEODOMAIN-LEUCINE ZIPPER III (HD-ZIP III) family [10]. To further test whether *ATHB8* promoted vein formation and to test whether *ATHB8* did so redundantly with other *HD-ZIP III* genes, we expressed *microRNA165a* (*miR165a*) — which targets all the *HD-ZIP III* genes [21] — by the *SHORT-ROOT* (*SHR*) promoter — which drives expression in the *ATHB8* expression domain [22] (Additional File 1: Fig. S1A–D) — in both the WT and *athb8-11* backgrounds.

The cardinality index of SHR::miR165a was lower than that of WT, and the cardinality index of SHR::miR165a;*athb8-11* was lower than that of SHR::miR165a (Fig. 1D,E,K), supporting that *ATHB8* promotes vein formation and suggesting that *ATHB8* does so redundantly with other *HD-ZIP III* genes.

HD-ZIP III proteins bind DNA as homo- or hetero-dimers [23, 24]. Therefore, to further test whether *ATHB8* promoted vein formation and whether *ATHB8* did so redundantly with other *HD-ZIP III* genes, we generated a dominant-negative version of the ATHB8 transcriptional activator [25] by fusing the *ATHB8* ORF to the sequence encoding the EAR (ethylene-responsive-element-binding-protein-associated amphiphilic repression) portable repressor domain [26]. In the resulting ATHB8:EAR, we introduced silent mutations that abolish *miR165a*-mediated downregulation [27]. We expressed the resulting mATHB8:EAR by the *SHR* promoter in both the WT and *athb8-27* backgrounds.

The cardinality index of SHR::mATHB8:EAR was lower than that of WT, and the cardinality index of SHR::mATHB8:EAR;*athb8-27* was lower than that of SHR::mATHB8:EAR (Fig. 1F,G,K), supporting that *ATHB8* promotes vein formation and that *ATHB8* does so redundantly with other *HD-ZIP III* genes.

We next asked whether levels of *ATHB8* expression and width of *ATHB8* expression domains were relevant to vein formation. To address this question, we used SHR::mATHB8, which overexpresses *ATHB8* in its expression domain; MP::ATHB8, which expresses *ATHB8* in the broader *MP* expression domain (Additional File 1: Fig. S1E); and MP::mATHB8, which overexpresses ATHB8 in the *MP* expression domain (Additional File 1: Fig. S1F).

The cardinality index of SHR::mATHB8 was lower than that of WT; the cardinality index of MP::ATHB8 was lower than that of SHR::mATHB8; and the cardinality index of MP::mATHB8 was lower than that of MP::ATHB8 (Fig. 1H–K). These results suggest that both levels of *ATHB8* expression and width of *ATHB8* expression domains are relevant to vein formation.

The continuity and connectivity indices of the genetic backgrounds with modified ATHB8 expression or activity either were no different from those of their respective reference backgrounds or changed with no consistent relation to changes in ATHB8 expression or activity (Fig. 1H–K). Therefore, the differences in cyclicity index of the genetic backgrounds with modified ATHB8 expression or activity can be attributed to differences in their cardinality index (Fig. 1H–K), from which the cyclicity index is derived (see “Methods”).

In the root, *HD-ZIP III* genes promote differentiation of the xylem vascular tissue [28, 29]. We therefore asked whether changes in ATHB8 expression or activity led to defects in leaf xylem differentiation.

Veins in SHR::miR165a had gaps in xylem differentiation, and those gaps were longer in SHR::miR165a;*athb8-11* (Fig. 1 L,O,P). By contrast, the veins of the remaining genetic backgrounds with modified ATHB8 expression or activity had no defects in xylem differentiation (Fig. 1 L–N,Q–U).

In conclusion, our results suggest that *ATHB8* promotes vein formation, both nonredundantly and redundantly with other *HD-ZIP III* genes; that levels of *ATHB8* expression and width of *ATHB8* expression domains are relevant to vein formation; and that *ATHB8* promotes xylem differentiation but only redundantly with other *HD-ZIP III* genes. By contrast, *ATHB8* is inconsequential to vein continuity and network connectedness.

### Relation between *ATHB8* expression domains and MP expression levels

Width of *ATHB8* expression domains is relevant to vein formation (Fig. 1). Therefore, we asked how *ATHB8* expression is activated in narrow preprocambial stripes by the broadly expressed MP. We hypothesized that *ATHB8* preprocambial expression is activated in narrow stripes by binding of peak levels of the broadly expressed MP to a low-affinity site in the *ATHB8* promoter. This hypothesis predicts that narrow stripes of *ATHB8* preprocambial expression correspond to peak levels of MP expression. To test this prediction, we simultaneously imaged expression of ATHB8::nCFP (nuclear CFP expressed by the *ATHB8* promoter) [14] and MP::MP:YFP (MP:YFP fusion protein expressed by the *MP* promoter) in first leaves of the strong *mp-B4149* mutant [30], whose defects were rescued by MP::MP:YFP expression (Additional File 1: Fig. S2A–C) (Additional File 2: Table S1) [14, 16, 19, 26, 27, 30–43].

**Fig. 2:**
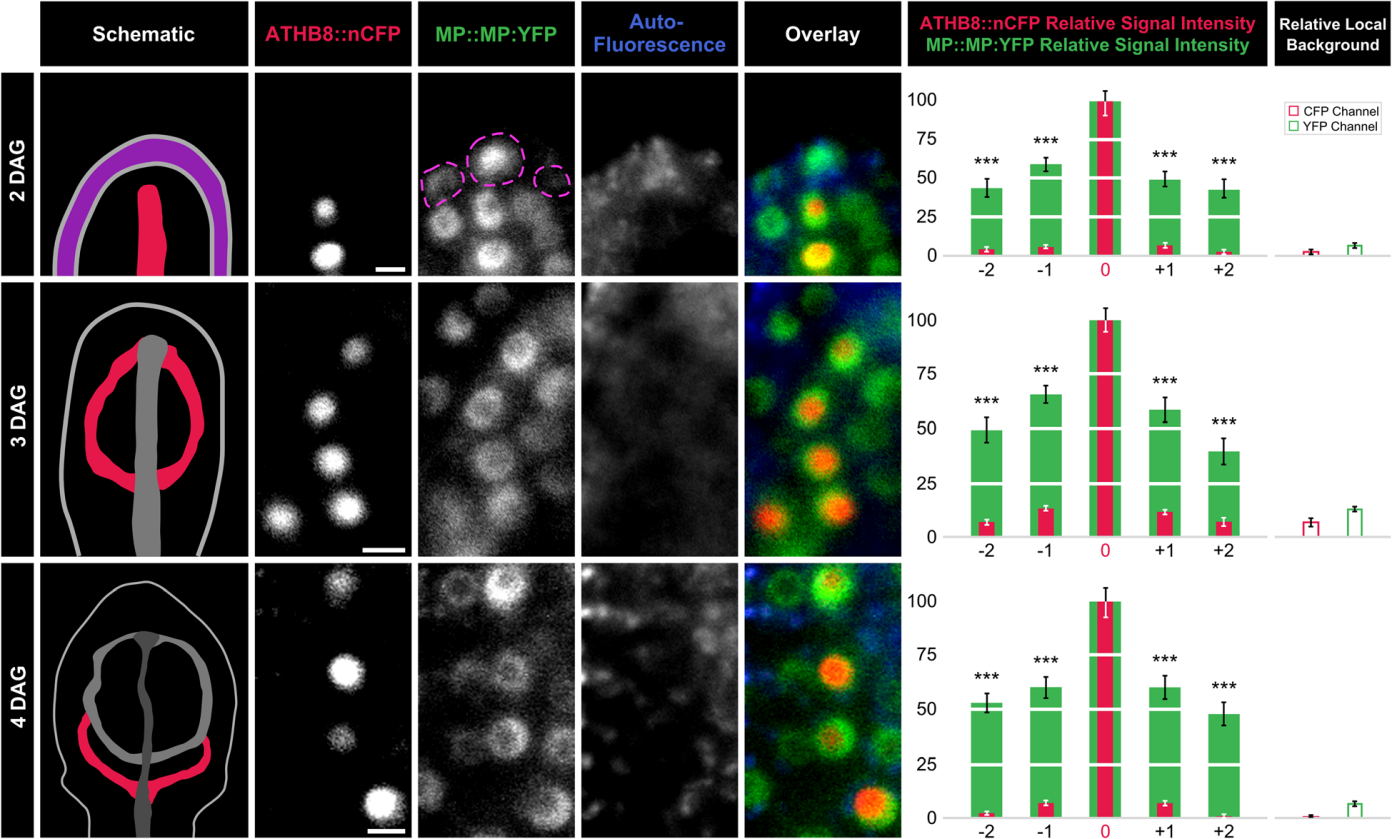
*ATHB8* and MP Expression Domains and Levels in Leaf Development. First leaves 2, 3, and 4 DAG. Column 1: schematics of leaves — imaged in columns 2–5 — illustrating onset of *ATHB8* expression (red) — imaged in column 2 — associated with formation of midvein (2 DAG), first loop (3 DAG), or second loop (4 DAG) [16, 22, 44]. Magenta: epidermis; increasingly darker gray: progressively older *ATHB8* expression domains. Columns 2–5: confocal laser scanning microscopy. Column 2: ATHB8::nCFP expression. Column 3: MP::MP:YFP expression; dashed magenta outline: MP::MP:YFP-expressing epidermal nuclei. Column 4: autofluorescence. Column 5: overlays of images in columns 2–4; red: ATHB8::nCFP expression; green: MP::MP:YFP expression; blue: autofluorescence. Column 6: ATHB8::nCFP and MP::MP:YFP expression levels (mean ± SE) in nuclei flanking ATHB8::nCFP-expressing nuclei (positions “-2”, “-1”, “*+*1”, and “*+*2”) relative to ATHB8::nCFP and MP::MP:YFP expression levels in nuclei co-expressing ATHB8::nCFP (position “0”) during formation of midvein (top), first loop (middle), or second loop (bottom). Difference between ATHB8::nCFP expression levels in nuclei at position -2, -1, *+*1, or *+*2 and ATHB8::nCFP expression levels in nuclei at position 0, and between MP::MP:YFP expression levels in nuclei at position -2, -1, *+*1, or *+*2 and MP::MP:YFP expression levels in nuclei at position 0 was significant at *P* *<* 0.001 (***) by One-Way ANOVA and Tukey’s Pairwise test. ATHB8::nCFP sample sizes: 35 (2 DAG), 33 (3 DAG), or 32 (4 DAG) leaves; position -2: 29 (2 DAG), 43 (3 DAG), or 49 (4 DAG) nuclei; position -1: 57 (2 DAG), 70 (3 DAG), or 66 (4 DAG) nuclei; position 0: 63 (2 DAG), 73 (3 DAG), or 69 (4 DAG) nuclei; position *+*1: 52 (2 DAG), 46 (3 DAG), or 58 (4 DAG) nuclei; position *+*2: 23 (2 DAG), 19 (3 DAG), or 37 (4 DAG) nuclei. MP::MP:YFP sample sizes: 35 (2 DAG), 33 (3 DAG), or 32 (4 DAG) leaves; position -2: 30 (2 DAG), 45 (3 DAG), or 50 (4 DAG) nuclei; position -1: 63 (2 DAG), 72 (3 DAG), or 67 (4 DAG) nuclei; position 0: 70 (2 DAG), 75 (3 DAG), or 70 (4 DAG) nuclei; position *+*1: 58 (2 DAG), 47 (3 DAG), or 59 (4 DAG) nuclei; position *+*2: 24 (2 DAG), 19 (3 DAG), or 38 (4 DAG) nuclei. Column 7: Local background levels (mean ± SE) in CFP and YFP confocal channels — measured in image regions containing no features of interest as in [14, 100] — relative to ATHB8::nCFP and MP::MP:YFP expression levels in nuclei co-expressing ATHB8::nCFP (position “0”) during formation of midvein (top), first loop (middle), or second loop (bottom). Scale bars (shown, for simplicity, only in column 2): 5 μm

*ATHB8* preprocambial expression can be reproducibly observed in midvein, first loops of veins (“first loops”), and second loops of first leaves, respectively 2, 3, and 4 days after germination (DAG) [16, 22, 44]. At these stages, MP::MP:YFP was expressed in ATHB8::nCFP-expressing cells at higher levels than in cells flanking ATHB8::nCFP-expressing cells (Fig. 2; Additional File 1: Fig. S3A,B).

To test whether the differential expression of MP::MP:YFP in ATHB8::nCFP-expressing cells and in cells flanking ATHB8::nCFP-expressing cells were an imaging artifact, we compared expression levels of nCFP driven by a ubiquitously active promoter (RIBO::nCFP) [31] in cells expressing ATHB8::nYFP [14] and in cells flanking ATHB8::nYFP-expressing cells. We focused our analysis on second loops of 4-DAG first leaves, in which *ATHB8* preprocambial expression can be reproducibly observed [16, 22, 44].

Because levels of RIBO::nCFP expression in ATHB8::nYFP-expressing cells were no higher than those in cells flanking ATHB8::nYFP-expressing cells (Additional File 1: Fig. S3D,E; Additional File 1: Figure S4), we conclude that the differential expression of MP::MP:YFP in ATHB8::nCFP-expressing cells and in cells flanking ATHB8::nCFP-expressing cells is not an imaging artifact, and therefore that narrow stripes of *ATHB8* preprocambial expression correspond to peak levels of MP expression.

### Response of *ATHB8* expression and vein network formation to changes in MP expression

The hypothesis — that *ATHB8* preprocambial expression is restricted to narrow stripes by binding of peak levels of the broadly expressed MP transcription factor to a low-affinity site in the *ATHB8* promoter — predicts that loss of *MP* function will lead to extremely weak, or altogether absent, *ATHB8* preprocambial expression, otherwise normally visible in second loops of 4-DAG first leaves [16, 22, 44]. To test this prediction, we quantified ATHB8::nYFP expression levels in second loops of 4-DAG first leaves of the strong *mp-U55* mutant [16, 32].

**Fig. 3:**
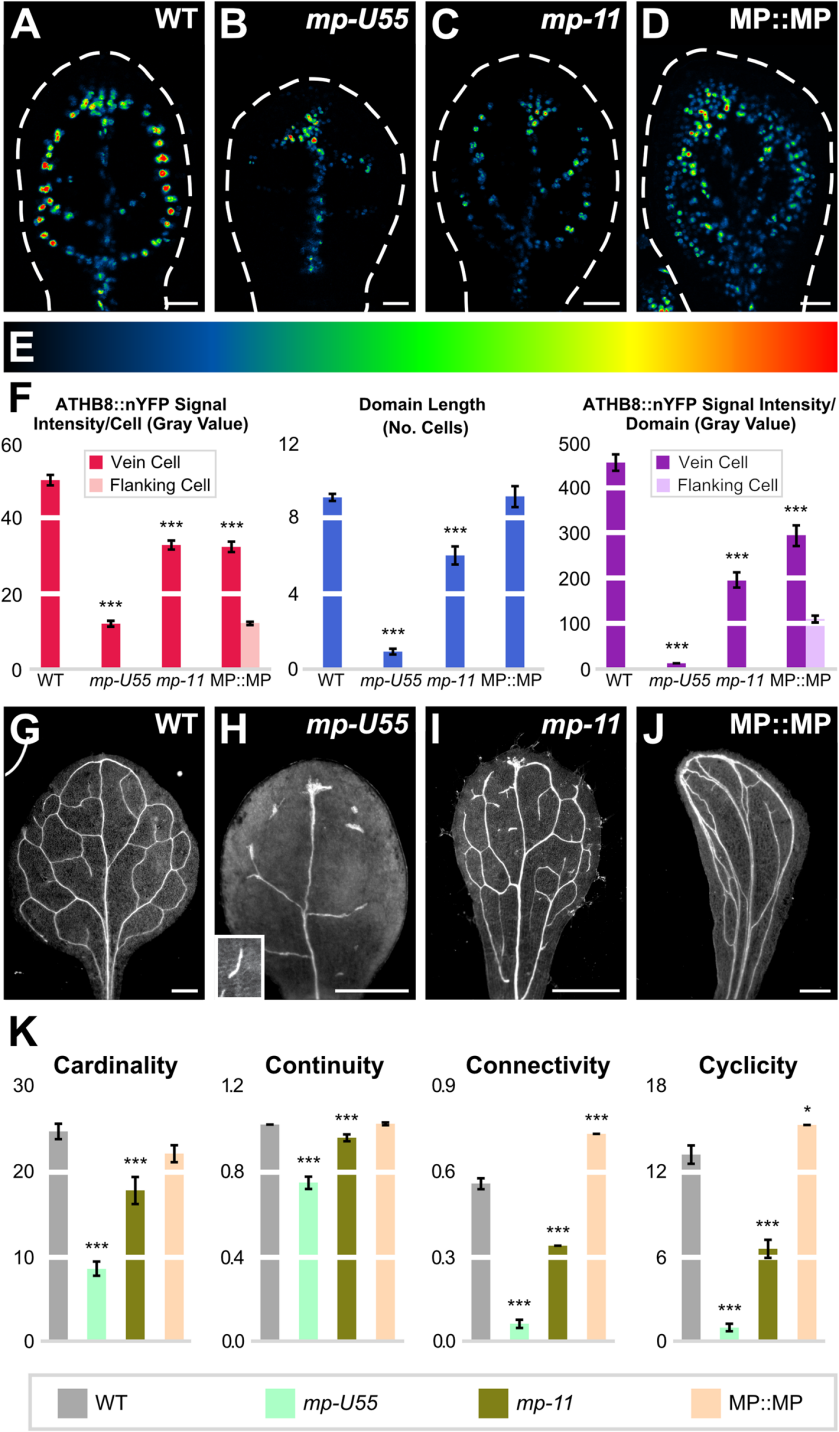
*MP* Expression, *ATHB8* Expression Domains and Levels, and Vein Network Formation. (**A**–**D**,**G**–**J**) Top right: genotype. (**A**–**D**) First leaves 4 DAG. Confocal laser scanning microscopy. Dashed white line: leaf outline. ATHB8::nYFP expression (look-up table — ramp in **E** — visualizes expression levels). (**F**) ATHB8::nYFP expression level per cell expressed as mean gray value ± SE, ATHB8::nYFP expression domain length expressed as mean number of cells ± SE, and ATHB8::nYFP expression levels per domain expressed as mean gray value ± SE. Difference between *mp-U55* and WT, between *mp-11* and WT, and between MP::MP and WT was significant at *P* *<* 0.001 (***) by *F*-test and *t*-test with Bonferroni correction. Sample sizes: 25 (WT), 72 (*mp-U55*), 27 (*mp-11*), or 24 (MP::MP) leaves; 345 (WT), 128 (*mp-U55*), 325 (*mp-11*), or 219 (MP::MP) vein cell nuclei, and 513 (MP::MP) flanking cell nuclei. (**G**–**J**) Dark-field illumination of cleared first leaves 14 DAG. (**K**) Cardinality index, connectivity index, and continuity index (mean ± SE) as defined in [20] and Methods; cyclicity index (mean ± SE) as defined in Methods. Difference between *mp-U55* and WT cardinality indices, between *mp-11* and WT cardinality indices, between *mp-U55* and WT continuity indices, between *mp-11* and WT continuity indices, between *mp-U55* and WT connectivity indices, between *mp-11* and WT connectivity indices, between MP::MP and WT connectivity indices, between *mp-U55* and WT cyclicity indices, between *mp-11* and WT cyclicity indices, and between MP::MP and WT cyclicity indices was significant at *P* *<* 0.05 (*) or *P* *<* 0.001 (***) by *F*-test and *t*-test with Bonferroni correction. Sample sizes: WT, 39; *mp-U55*, 59; *mp-11*, 44; MP::MP, 41. Scale bars: (**A**–**D**) 25 μm; (**G**-**J**) 0.5 mm

Consistent with previous observations [16], ATHB8::nYFP expression levels were greatly reduced in *mp-U55*, leading to near-complete loss of ATHB8::nYFP preprocambial expression (Fig. 3A,B,F). Moreover, consistent with previous observations [16, 17], near-complete loss of *ATHB8* preprocambial expression in *mp-U55* developing leaves was associated with networks of fewer meshes and fewer, less frequently continuous, and less frequently connected veins in *mp-U55* mature leaves (Fig. 3G,H,K).

The hypothesis further predicts that lower levels of *MP* expression will lead to lower levels of *ATHB8* preprocambial expression. To test this prediction, we quantified ATHB8::nYFP expression levels in second loops of 4-DAG first leaves of the weak *mp-11* mutant, in which an insertion in the *MP* promoter [33] leads to ~85% reduction in levels of WT *MP* transcript (Additional File 1: Figure S5).

In *mp-11*, ATHB8::nYFP expression levels were lower and expression along the domain was more heterogeneous than in WT, leading to seemingly fragmented domains of weak ATHB8::nYFP preprocambial expression (Fig. 3A,C,F). Moreover, as in *mp-U55*, defects in *ATHB8* expression in *mp-11* developing leaves were associated with networks of fewer meshes and fewer, less frequently continuous, and less frequently connected veins in *mp-11* mature leaves (Fig. 3G,I,K). However, the vein network and *ATHB8* expression defects of *mp-11* were weaker than those of *mp-U55* (Fig. 3A–C,G–I,K).

The hypothesis also predicts that higher levels of the broadly expressed MP will lead to higher levels of *ATHB8* preprocambial expression in both vein and flanking cells, resulting in broader *ATHB8* expression domains. To test this prediction, we overexpressed *MP* by its own promoter (MP::MP) — which led to ~10-fold increase in *MP* expression levels (Additional File 1: Figure S5) and which rescued defects of the strong *mp-B4149* mutant (Additional File 1: Fig. S2A,B,D) (Additional File 2: Table S1) — and quantified ATHB8::nYFP expression levels in second loops of 4-DAG MP::MP first leaves.

In MP::MP, ATHB8::nYFP expression levels were higher in flanking cells, leading to broad bands of ATHB8::nYFP expression; however, ATHB8::nYFP expression levels were lower in vein cells (Fig. 3A,D,F). Nevertheless, broad bands of *ATHB8* expression in MP::MP developing leaves were associated with abnormal vein networks in MP::MP mature leaves: veins ran close to one another for varying stretches of the narrow leaf laminae, then diverged, and either ran close to other veins or converged back to give rise to elongated meshes (Fig. 3G,J,K).

In conclusion, lower levels of *MP* expression lead to fragmented domains of *ATHB8* preprocambial expression, and loss of *MP* function leads to near-complete loss of *ATHB8* preprocambial expression. These observations are consistent with the hypothesis and suggest that *MP* expression levels below a minimum threshold are unable to activate *ATHB8* preprocambial expression. However, that higher levels of *MP* expression fail to lead to higher levels of *ATHB8* preprocambial expression in vein cells is inconsistent with the hypothesis and suggests that *MP* expression levels above a maximum threshold both activate and repress *ATHB8* preprocambial expression. These observations are unaccounted for by the hypothesis; therefore, the hypothesis must be revised.

### Response of *ATHB8* expression and vein network formation to changes in MP activity

*MP* expression levels above a maximum threshold both activate and repress *ATHB8* preprocambial expression (Fig. 3). Activation of *ATHB8* preprocambial expression by MP is direct [16], but repression of *ATHB8* preprocambial expression by MP need not be: *MP*-dependent repression of *ATHB8* preprocambial expression could be mediated, for example, by an AUXIN/INDOLE-3-ACETIC-ACID-INDUCIBLE (AUX/IAA) protein such as BODENLOS (BDL)/IAA12 (BDL hereafter), whose expression is activated by MP and which binds to MP and inhibits its transcriptional activity [30, 45–47]. Were *MP*-dependent repression of *ATHB8* preprocambial expression mediated by BDL, *ATHB8* preprocambial expression would be reduced in the *bdl* mutant, in which the unstable BDL protein is stabilized [41]. To test this prediction, we quantified ATHB8::nYFP expression levels in second loops of 4-DAG first leaves of the *bdl* mutant.

**Fig. 4:**
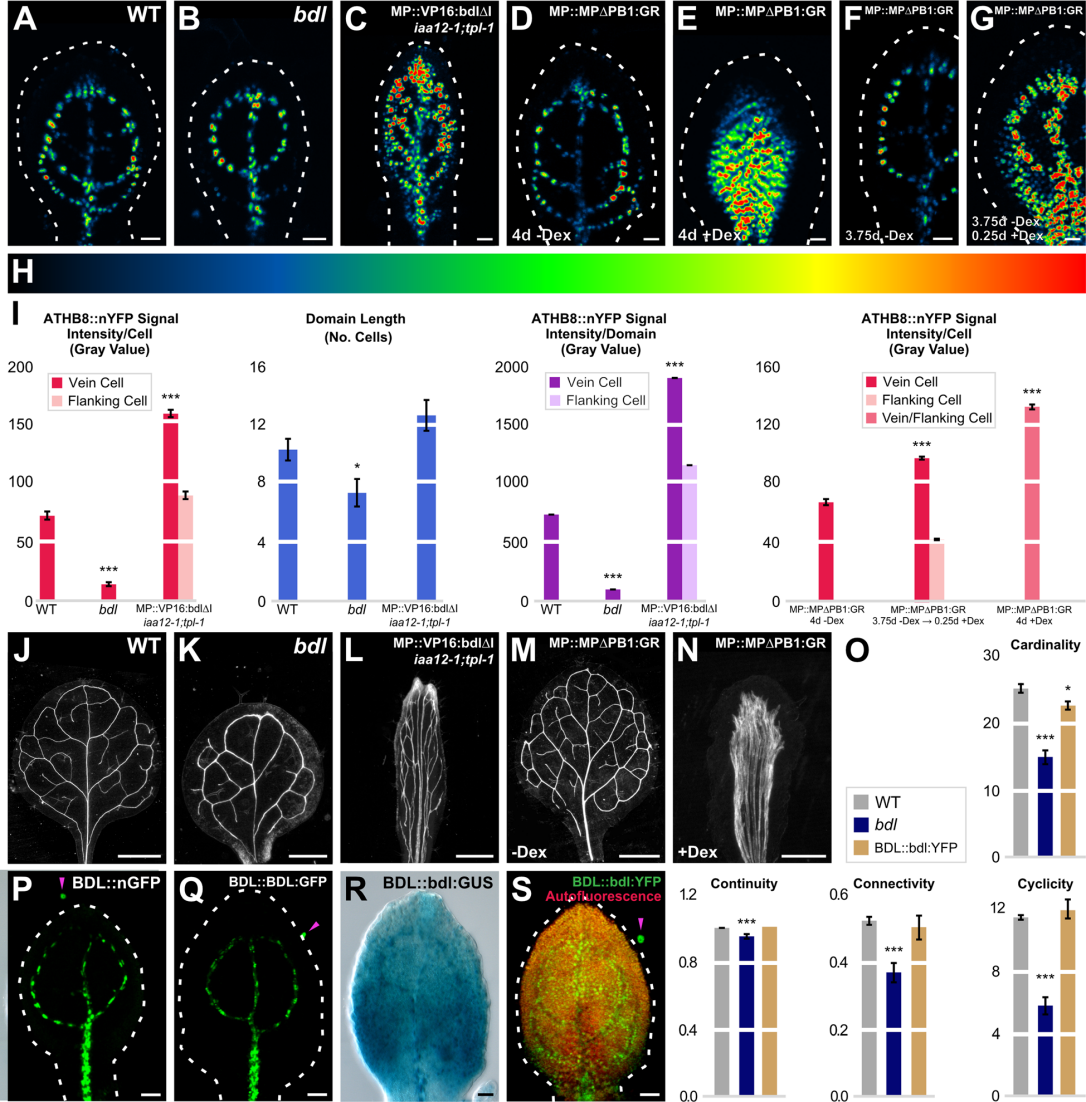
MP Activity, *ATHB8* Expression Domains and Levels, and Vein Network Formation. (**A**–**G**,**J**–**N**,**P**–**S**) Top right: genotype. (**D**–**G**,**M**,**N**) Bottom left: treatment. (**A**–**G**,**P**–**S**) First leaves 3.75 (**F**) or 4 (**A**–**E**,**G**,**P**–**S**) DAG (for simplicity, only half-leaves are shown in **F** and **G**). Confocal laser scanning (**A**–**G**,**P**,**Q**,**S**) or differential interference contrast (**R**) microscopy. Dashed white line: leaf outline. (**A**–**G**) ATHB8::nYFP expression (look-up table — ramp in **H** — visualizes expression levels). (**I**) ATHB8::nYFP expression level per cell expressed as mean gray value ± SE, ATHB8::nYFP expression domain length expressed as mean number of cells ± SE, and ATHB8::nYFP expression levels per domain expressed as mean gray value ± SE. Difference between *bdl* and WT, between MP::VP16:bdlΔI;*iaa12-1*;*tpl-1* and WT, between MP::MPΔPB1:GR 3.75d -Dex → 0.25d +Dex and MP::MPΔPB1:GR 4d -Dex, and between MP::MPΔPB1:GR 4d +Dex and MP::MPΔPB1:GR 4d -Dex was significant at *P* *<* 0.05 (*) or *P* *<* 0.001 (***) by *F*-test and *t*-test with Bonferroni correction. Sample sizes: 26 (WT), 27 (*bdl*), 27 (MP::VP16:bdlΔI;*iaa12-1*;*tpl-1*), 18 (MP::MPΔPB1:GR 4d -Dex), 27 (MP::MPΔPB1:GR 3.75d -Dex → 0.25d +Dex), or 19 (MP::MPΔPB1:GR 4d +Dex) leaves; 265 (WT), 199 (*bdl*), 338 (MP::VP16:bdlΔI;*iaa12-1*;*tpl-1*), 248 (MP::MPΔPB1:GR 4d -Dex), 284 (MP::MPΔPB1:GR 3.75d -Dex → 0.25d +Dex), or 269 (dex-grown MP::MPΔPB1:GR) vein cell nuclei, and 316 (MP::VP16:bdlΔI;*iaa12-1*;*tpl-1*) or 608 (MP::MPΔPB1:GR 3.75d -Dex → 0.25d +Dex) flanking cell nuclei. (**J**-**N**) Dark-field illumination of cleared first leaves 14 DAG. (**O**) Cardinality index, connectivity index, and continuity index (mean ± SE) as defined in [20] and Methods; cyclicity index (mean ± SE) as defined in Methods. Difference between *bdl* and WT cardinality indices, between BDL::bdl:YFP and WT cardinality indices, between *bdl* and WT continuity indices, between *bdl* and WT connectivity indices, and between *bdl* and WT cyclicity indices, was significant at *P* *<* 0.05 (*) or *P* *<* 0.001 (***) by *F*-test and *t*-test with Bonferroni correction. (**J**–**S**) Sample sizes: WT, 30; *bdl*, 65; MP::VP16:bdlΔI;*iaa12-1*;*tpl-1*, 22; MP::MPΔPB1:GR, 42; dex-grown MP::MPΔPB1:GR, 38; BDL::bdl:YFP (**O**), 20; BDL::nGFP, 46; BDL::BDL:GFP, 24; BDL::bdl:GUS, 32; BDL::bdl:YFP (**S**), 39. Scale bars: (**A**–**G**,**P**–**S**) 25 μm; (**J**,**M**,**N**) 1 mm; (**K**) 0.25 mm; (**L**) 0.5 mm

As in *mp*, in *bdl* levels of ATHB8::nYFP preprocambial expression levels were lower and expression along the domain was more heterogeneous than in WT, leading to seemingly fragmented domains of weak ATHB8::nYFP preprocambial expression (Fig. 3A–C,F; Fig. 4A,B,I). Moreover, as in *mp*, defects in *ATHB8* expression in *bdl* developing leaves were associated with networks of fewer meshes and fewer, less frequently continuous, and less frequently connected veins in *bdl* mature leaves (Fig. 3G–I,K; Fig. 4 J,K,O).

Were *MP*-dependent repression of *ATHB8* preprocambial expression mediated by an AUX/IAA protein such as BDL, reducing or eliminating AUX/IAA-mediated inhibition of MP transcriptional activity would lead to higher levels of *ATHB8* preprocambial expression in both vein and flanking cells, resulting in broader *ATHB8* expression domains. To test this prediction, we turned the unstable BDL transcriptional repressor into a stabilized transcriptional activator as previously done for other AUX/IAA proteins [48–50]: we replaced the repressor domain of BDL [51] with the activator domain of the *Herpes simplex* Virus Protein 16 (VP16) [35] and introduced a mutation that lengthens the half-life of BDL [45]. We expressed the resulting VP16:bdlΔI by the *MP* promoter in the *iaa12-1* mutant, which lacks *BDL* function [36], and the *iaa12-1*;*tpl-1* double mutant, which in addition partially lacks the co-repressor function that mediates the AUX/IAA-protein-dependent repression of MP [52]. We quantified ATHB8::nYFP expression levels in second loops of 4-DAG first leaves of the resulting MP::VP16:bdlΔI;*iaa12-1*;*tpl-1* background.

As in MP::MP, in both MP::VP16:bdlΔI;*iaa12-1* and MP::VP16:bdlΔI;*iaa12-1*;*tpl-1* — but not in *iaa12-1* — ATHB8::nYFP expression levels were higher in flanking cells (Fig. 3A,D,F; Fig. 4A,C,I; Additional File 1: Figure S6). Unlike in MP::MP, however, in both MP::VP16:bdlΔI;*iaa12-1* and MP::VP16:bdlΔI;*iaa12-1*;*tpl-1*, ATHB8::nYFP expression levels were also higher in vein cells (Fig. 3A,D,F; Fig. 4A,C,I; Additional File 1: Figure S6). Accordingly, stronger *ATHB8* expression domains in MP::VP16:bdlΔI;*iaa12-1*;*tpl-1* developing leaves were associated with stronger — though qualitatively similar — vein network defects in MP::VP16:bdlΔI;*iaa12-1*;*tpl-1* mature leaves: in the middle of these leaves, veins ran parallel to one another for the entire length of the narrow leaf laminae to give rise to wide midveins; toward the margin, veins ran close to one another for varying stretches of the laminae, then diverged, and either ran close to other veins or converged back to give rise to elongated meshes (Fig. 3G,J,K; Fig. 4J,L).

Next, we further tested the prediction that reducing or eliminating AUX/IAA-mediated inhibition of MP transcriptional activity would lead to higher levels of *ATHB8* preprocambial expression in both vein and flanking cells, resulting in broader *ATHB8* expression domains. As previously done [29, 53, 54], we created an irrepressible version of MP by deleting its PHOX/BEM1 (PB1) domain, which is required for AUX/IAA-mediated inhibition [49, 53, 55, 56]. We fused the resulting MPΔPB1 to a fragment of the rat glucocorticoid receptor (GR) [57] to confer dexamethasone (dex)-inducibility, expressed the resulting MPΔPB1:GR by the *MP* promoter, and quantified ATHB8::nYFP expression levels in 4-DAG first leaves of the dex-grown MP::MPΔPB1:GR background.

Consistent with previous observations [53, 58], in dex-grown MP::MPΔPB1:GR, ATHB8::nYFP expression was no longer restricted to narrow stripes; instead, ATHB8::nYFP was expressed at higher levels in broad bands than spanned almost the entire width of the leaves (Fig. 4D,E,I). Accordingly, broader and stronger *ATHB8* expression domains in dex-grown MP::MPΔPB1:GR developing leaves were associated with veins running parallel to one another for the entire length of the narrow leaf laminae to give rise to midveins that spanned almost the entire width of dex-grown MP::MPΔPB1:GR mature leaves (Fig. 4M–O).

Broader and stronger *ATHB8* expression domains in dex-grown MP::MPΔPB1:GR leaves may be the result of the leaves’ vein pattern defects, rather than of the reduction in AUX/IAA-mediated inhibition of *MP*-dependent activation of *ATHB8* expression. To test this possibility, we leveraged two observations: (1) *ATHB8* preprocambial expression is activated asynchronously in second loops during leaf development [13]; (2) by the time a vein has activated *ATHB8* preprocambial expression, the vein’s position has been specified [59]. We therefore germinated and grew ATHB8::nYFP;MP::MPΔPB1:GR in the absence of dex for 3.75 days, transferred the seedlings to dex-containing medium for 6 h, and quantified ATHB8::nYFP expression levels in the newly formed second loops of 4-DAG first leaves. Because in 3.75-DAG first leaves, *ATHB8* is expressed in midvein, first loops, and only one of the two second loops (Fig. 4F), the position of those veins can no longer be changed by dex-mediated activation of MP::MPΔPB1:GR. As such, any activation of *ATHB8* expression in the second loops formed after the dex-mediated activation of MP::MPΔPB1:GR would only be the result of the reduction in AUX/IAA-mediated inhibition of *MP*-dependent activation of *ATHB8* expression.

Consistent with what shown above (Fig. 4D,E,I), ATHB8::nYFP expression in the second loops formed after the dex-mediated activation of MP::MPΔPB1:GR was no longer restricted to narrow stripes; instead, ATHB8::nYFP was expressed at higher levels in broad bands (Fig. 4F,G,I). These results are consistent with the interpretation that broader and stronger *ATHB8* expression domains in dex-grown MP::MPΔPB1:GR leaves (Fig. 4D,E,I) are the result of the reduction in AUX/IAA-mediated inhibition of *MP*-dependent activation of *ATHB8* expression, rather than of the leaves’ vein pattern defects.

Our results suggest that *MP*-dependent repression of *ATHB8* preprocambial expression is mediated by AUX/IAA proteins, including BDL (Fig. 4A–G,I). However, that BDL mediates *MP*-dependent repression of *ATHB8* preprocambial expression is based upon the assumption that the lower levels of *ATHB8* preprocambial expression in the dominant *bdl* mutant reflect hypermorphic, as opposed to neomorphic, effects of the *bdl* mutation. Were BDL indeed mediating *MP*-dependent repression of *ATHB8* preprocambial expression, BDL expression domains would overlap with domains of *ATHB8* preprocambial expression. To test whether that were so, we imaged expression of BDL::nGFP and BDL::BDL:GFP in 4-DAG first leaves.

Contrary to expectations, BDL::nGFP and BDL::BDL:GFP were only expressed in midvein and first loops and were expressed neither in second loops nor in their flanking cells (Fig. 4P,Q). We therefore asked whether the *bdl* mutation affected BDL expression. To address this question, we imaged GUS activity in 4-DAG first leaves of a BDL::bdl:GUS line that recapitulates the *bdl* phenotype [41, 60].

BDL::bdl:GUS was strongly expressed in midvein and first loops; in the top half of the leaf, BDL::bdl:GUS was also expressed in the inner nonvascular tissue, though expression was weaker than in midvein and first loops (Fig. 4R). In the bottom half of the leaf, BDL::bdl:GUS was strongly expressed in both epidermis and inner tissue, including the areas where second loops were forming.

Broad expression of BDL::bdl:GUS may be the result of the leaves’ vein network defects, rather than of an effect of the *bdl* mutation on BDL expression. To test this possibility, we generated a BDL::bdl:YFP line that expresses the transgene at low levels and that therefore leads to only very minor vein network defects (Fig. 4O). We then imaged expression of BDL::bdl:YFP in 4-DAG first leaves.

The expression of BDL::bdl:YFP mirrored that of BDL::bdl:GUS, including expression in second loops and their flanking cells (Fig. 4S), suggesting that broad expression of BDL::bdl:GUS is the result of an effect of the *bdl* mutation on BDL expression, rather than of the leaves’ vein network defects. Moreover, these observations suggest neomorphic, as opposed to hypermorphic, effects of the *bdl* mutation on *ATHB8* preprocambial expression.

In conclusion, our results are consistent with the hypothesis that *MP* expression levels above a maximum threshold both activate and repress *ATHB8* preprocambial expression and that such *MP*-dependent repression of *ATHB8* preprocambial expression is mediated by AUX/IAA proteins; such AUX/IAA proteins, however, are unlikely to include BDL.

### Relation between *ATHB8* expression domains and auxin levels

AUX/IAA proteins are degraded in response to the plant hormone auxin [41, 48, 61, 62]. Auxin-dependent degradation of AUX/IAA proteins releases MP from inhibition, thus allowing MP to activate expression of its targets, including *AUX*/*IAA* genes and *ATHB8* [16, 30, 46, 47, 53, 58, 63–67]. Therefore, narrow stripes of *ATHB8* preprocambial expression should correspond to peak levels of sensed auxin. To test this prediction, we simultaneously imaged in midvein, first loops, and second loops of developing first leaves expression of ATHB8::nQFP (nuclear Turquoise Fluorescent Protein expressed by the *ATHB8* promoter) and of the auxin ratiometric reporter R2D2 [42], which expresses an auxin-degradable nYFP and a non-auxin-degradable nRFP by the *RIBOSOMAL PROTEIN S5A* promoter, which is highly active in developing leaves [68]. In the R2D2 reporter, a high RFP/YFP ratio thus indicates high levels of auxin, whereas a low RFP/YFP ratio indicates low levels of auxin [42].

**Fig. 5:**
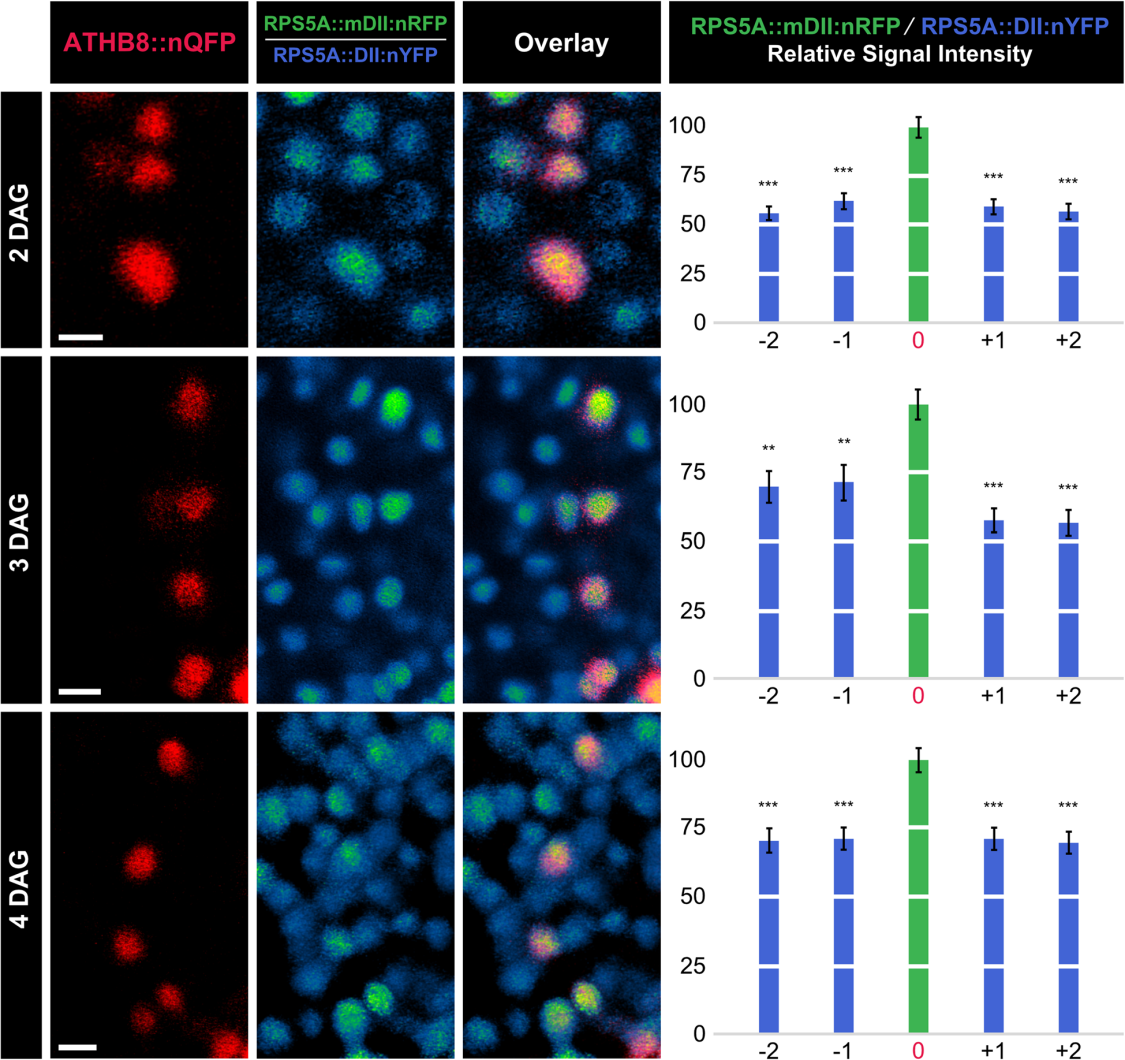
*ATHB8* Expression Domains and Auxin Levels. First leaves 2, 3, and 4 DAG. Columns 1–3: confocal laser scanning microscopy. Column 1: ATHB8::nQFP expression (red) associated with formation of midvein (2 DAG), first loop (3 DAG), or second loop (4 DAG) [16, 22, 44]. Column 2: Ratio of RPS5A::mDII:nRFP expression to RPS5A::DII:nYFP expression. Look-up table visualizes expression ratio levels: high RPS5A::mDII:nRFP/RPS5A::DII:nYFP ratio (green) indicates high auxin levels; low RPS5A::mDII:nRFP/RPS5A::DII:nYFP ratio (blue) indicates low auxin levels. Column 3: overlays of images in columns 1 and 2; blue: low RPS5A::mDII:nRFP/RPS5A::DII:nYFP ratio, i.e. low auxin levels; yellow: co-expression of ATHB8::nQFP (red) and high RPS5A::mDII:nRFP/RPS5A::DII:nYFP ratio (green), i.e. high auxin levels. Column 4: Ratio of RPS5A::mDII:nRFP expression levels to RPS5A::DII:nYFP expression levels (mean ± SE) in nuclei flanking ATHB8::nQFP-expressing nuclei (positions “-2”, “-1”, “*+*1”, and “*+*2”) relative to ratio of RPS5A::mDII:nRFP expression levels to RPS5A::DII:nYFP expression levels in nuclei co-expressing ATHB8::nQFP (position “0”) during formation of midvein (top), first loop (middle), or second loop (bottom). Difference between ratio of RPS5A::mDII:nRFP expression levels to RPS5A::DII:nYFP expression levels in nuclei at position -2, -1, *+*1, or *+*2 and ratio of RPS5A::mDII:nRFP expression levels to RPS5A::DII:nYFP expression levels in nuclei at position 0 was significant at *P* *<* 0.01 (**) or *P* *<* 0.001 (***) by One-Way ANOVA and Tukey’s Pairwise test. Sample sizes: 26 (2 DAG), 27 (3 DAG), or 29 (4 DAG) leaves; position -2: 56 (2 DAG), 42 (3 DAG), or 60 (4 DAG) nuclei; position -1: 52 (2 DAG), 37 (3 DAG), or 58 (4 DAG) nuclei; position 0: 74 (2 DAG), 85 (3 DAG), or 102 (4 DAG) nuclei; position *+*1: 44 (2 DAG), 44 (3 DAG), or 62 (4 DAG) nuclei; position *+*2: 42 (2 DAG), 25 (3 DAG), or 44 (4 DAG) nuclei. Scale bars (shown, for simplicity, only in column 2): 5 μm

At all tested stages, the RFP/YFP ratio was higher in ATHB8::nQFP-expressing cells than in cells flanking ATHB8::nQFP-expressing cells (Fig. 5), suggesting that domains of *ATHB8* preprocambial expression correspond to peak levels of sensed auxin.

### Response of *ATHB8* expression to manipulation of MP-binding site affinity

The hypothesis that *MP* expression levels below a minimum threshold are unable to activate *ATHB8* preprocambial expression predicts that reducing the affinity of MP for its binding site in the *ATHB8* promoter will lead to extremely weak, or altogether absent, *ATHB8* preprocambial expression.

To test this prediction, we mutated the MP-binding site in the *ATHB8* promoter (TGTCTG) to lower (TGTCAG) or negligible (TAGCTG) affinity for MP binding [16, 69–71], and imaged nYFP expressed by the native or mutant promoters in second loops of 4-DAG first leaves.

**Fig. 6:**
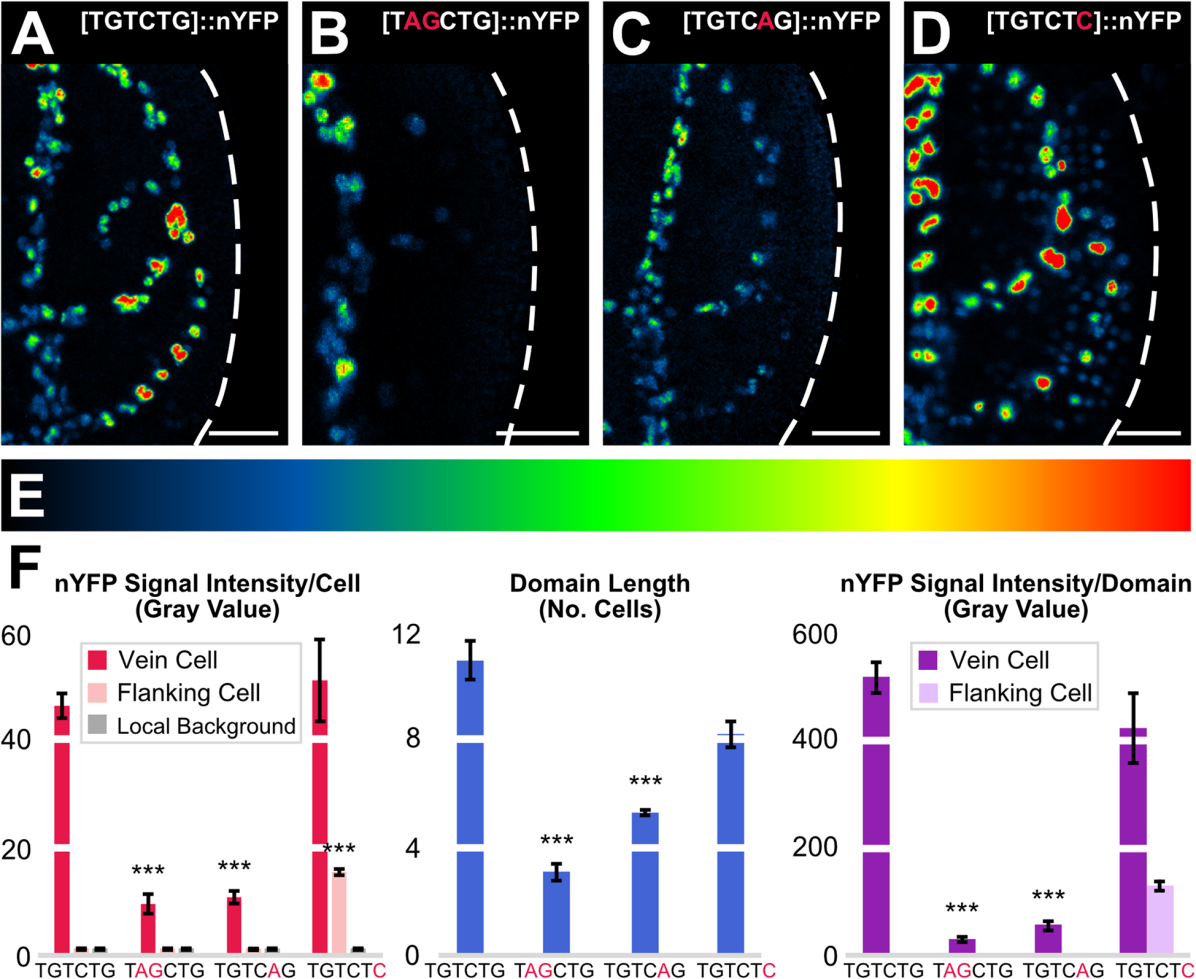
Activity of *ATHB8* Promoter Variants. (**A**–**D**) First leaves 4 DAG (for simplicity, only half-leaves are shown). Confocal laser scanning microscopy. nYFP expression (look-up table — ramp in **E** — visualizes expression levels) driven by promoter variants (top right) with native ([TGTCTG]::nYFP, i.e. ATHB8::nYFP) (**A**), negligible ([TAGCTG]::nYFP) (**B**), lower ([TGTCAG]::nYFP) (**C**), or higher ([TGTCTC]::nYFP) (**D**) affinity for MP binding. Dashed white line: leaf outline. (**F**) nYFP expression level per cell expressed as mean gray value ± SE, nYFP expression domain length expressed as mean number of cells ± SE, and nYFP expression level per domain expressed as mean gray value ± SE. Local background levels were measured in image regions containing no features of interest as in [14, 100]. Difference between [TAGCTG]::nYFP expression levels in vein cell nuclei and [TGTCTG]::nYFP expression levels in vein cell nuclei, between [TGTCAG]::nYFP expression levels in vein cell nuclei and [TGTCTG]::nYFP expression levels in vein cell nuclei, and between [TGTCTC]::nYFP expression levels in flanking cell nuclei and [TGTCTG]::nYFP expression levels in flanking cell nuclei was significant at *P* *<* 0.001 (***) by *F*-test and *t*-test with Bonferroni correction. Sample sizes: 31 ([TGTCTG]::nYFP), 32 ([TAGCTG]::nYFP), 38 ([TGTCAG]::nYFP), or 35 ([TGTCTC]:nYFP) leaves; 538 ([TGTCTG]::nYFP), 91 ([TAGCTG]::nYFP), 296 ([TGTCAG]::nYFP), or 420 ([TGTCTC]::nYFP) vein cell nuclei, and 328 ([TGTCTG]::nYFP), 175 ([TAGCTG]::nYFP), 398 ([TGTCAG]::nYFP), or 1,144 ([TGTCTC]::nYFP) flanking cell nuclei. In [TGTCTG]::nYFP, [TAGCTG]::nYFP, and [TGTCAG]::nYFP, flanking cell nuclei were identified by means of RIBO::nCFP expression. In [TGTCTC]::nYFP, vein cell nuclei were identified by means of ATHB8::nCFP expression. Scale bars: 25 μm

Mutation of the MP-binding site in the *ATHB8* promoter to negligible affinity for MP binding led to greatly reduced levels of nYFP expression (Fig. 6A,B,F), resembling near-complete loss of ATHB8::nYFP preprocambial expression in *mp-U55* [16] (Fig. 3A,B,F). Mutation of the MP-binding site in the *ATHB8* promoter to lower affinity for MP binding led to lower levels of nYFP expression (Fig. 6A,C,F). Furthermore, expression along the domains was more heterogeneous than when nYFP was expressed by the native promoter (Fig. 6A,C,F), leading to seemingly fragmented domains of weak nYFP expression similar to those in *mp-11* (Fig. 3A,C,F) and *bdl* (Fig. 4A,B,I).

The hypothesis that *MP* expression levels above a maximum threshold both activate and repress *ATHB8* preprocambial expression predicts that increasing the affinity of MP for its binding site in the *ATHB8* promoter will lead to higher levels of *ATHB8* preprocambial expression in flanking cells, leading to broader *ATHB8* expression domains, and to levels of *ATHB8* preprocambial expression in vein cells that are no lower — though not necessarily any higher — than those in WT.

To test this prediction, we mutated the MP-binding site in the *ATHB8* promoter (TGTCTG) to higher (TGTCTC) affinity for MP binding [16, 69, 70], and imaged nYFP expressed by the native or mutant promoter in second loops of 4-DAG first leaves.

Mutation of the MP-binding site in the *ATHB8* promoter to higher affinity for MP binding led to higher levels of nYFP expression in flanking cells (Fig. 6A,D,F), resulting in broad bands of nYFP expression similar to those in MP::MP (Fig. 3A,D,F) and, to a lesser extent, MP::VP16:bdlΔI;*iaa12-1* (Additional File 1: Fig. S6B), MP::VP16:bdlΔI;*iaa12-1*;*tpl-1* (Fig. 4A,C,I), and dex-grown MP::MPΔPB1:GR (Fig. 4D–G,I). However, unlike in MP::MP — in which ATHB8::nYFP expression levels in vein cells were lower than in WT (Fig. 3A,D,F) — and unlike in MP::VP16:bdlΔI;*iaa12-1*, MP::VP16:bdlΔI;*iaa12-1*;*tpl-1*, and dex-grown MP::MPΔPB1:GR — in which those levels were higher (Fig. 4A,C–G,I; Additional File 1: Fig. S6A,B) — nYFP expression levels in vein cells were unchanged by mutation of the MP-binding site in the *ATHB8* promoter to higher affinity for MP binding (Fig. 6A,D,F), suggesting that MP levels are normally nonlimiting for *ATHB8* preprocambial expression.

In conclusion, our results are consistent with the hypothesis that *MP* expression levels below a minimum threshold are unable to activate *ATHB8* preprocambial expression and that *MP* expression levels above a maximum threshold both activate and repress *ATHB8* preprocambial expression.

## Discussion

A long-standing problem in biology is how gene expression is activated in narrow stripes by broadly expressed transcription factors (e.g., [72, 73]). Here we addressed this problem for plants by means of the *MP* – *ATHB8* pair of Arabidopsis genes.

Consistent with interpretation of similar findings in animals (e.g., [74–76]), our results suggest that levels of expression of the MP transcription factor above a maximum threshold both activate and repress *ATHB8* preprocambial expression. *MP*-dependent activation of *ATHB8* expression is direct [16] and — we found — mediated by binding of MP to a low-affinity site in the *ATHB8* promoter. By contrast, we found that *MP*-dependent repression of *ATHB8* expression is indirect and mediated by members of the *AUX*/*IAA* family, which are themselves direct targets of MP [47, 64]. AUX/IAA proteins inhibit MP transcriptional activity and are degraded at peak levels of the plant hormone auxin [30, 41, 46, 48, 53, 58, 61–63, 66, 67] such as those we found corresponding to narrow stripes of *ATHB8* preprocambial expression. As such, our results suggest that an incoherent type-I feedforward loop [77] restricts activation of *ATHB8* preprocambial expression to narrow stripes: auxin activates *MP*, which in turn activates expression of intermediate-loop *AUX*/*IAA* genes; and *MP* and *AUX*/*IAA* genes jointly regulate expression of *ATHB8*, which converts the auxin signal input into vein formation output (Additional File 1: Figure S7).

Our finding that *ATHB8* promotes vein formation both nonredundantly and redundantly with other *HD-ZIP III* genes is consistent with the observation that excess vein formation in the *acaulis5* mutant depends on the function of *ATHB8* and of the *ATHB8*-related *REVOLUTA* and *ATHB15*/*CORONA* genes [25]. Nevertheless, precisely how *ATHB8* promotes vein formation remains unclear. Delayed vein formation in *athb8* mutants [16] suggests that *ATHB8* promotes timely vein formation, possibly preventing premature termination of initiation of vein formation by mesophyll differentiation [13]. Furthermore, because the *athb8* mutation enhances the defects in coordination of cell polarity and vein patterning induced by the inhibition of the polar, cell-to-cell transport of auxin [16], it is possible that *ATHB8* belongs to that auxin signaling pathway that controls coordination of cell polarity and vein patterning redundantly with polar auxin transport [78]. However, these possibilities remain to be tested.

Given the defects in *ATHB8* preprocambial expression we observed in the *bdl* mutant, our finding that the AUX/IAA protein BDL is unlikely to be mediating *MP*-dependent repression of *ATHB8* preprocambial expression is perhaps unexpected but certainly not unprecedented. Not only in veins — as we found — but in embryos too, the *bdl* mutation leads to expression of the bdl protein at stages earlier and in domains broader than those at and in which the BDL protein is expressed [63]. Furthermore, a mutation in the *CRANE*/*IAA18* gene that, just like the *bdl* mutation, stabilizes the resulting mutant protein also leads to expression of the crane-2/iaa18-1 mutant protein at stages earlier and in domains broader than those at and in which the CRANE/IAA18 protein is expressed [66, 79]. These observations reinforce the need for caution when interpreting phenotypes of dominant mutants as hypermorphic — as opposed to neomorphic — as it has often been done for dominant *aux*/*iaa* mutations. In the future, it will be interesting to identify which AUX/IAA proteins mediate *MP*-dependent repression of *ATHB8* preprocambial expression; as interesting as that identification will be, however, it will also be unlikely to change the logic of the regulatory network that we propose restricts *ATHB8* preprocambial expression to narrow stripes.

In the future, it will also be interesting to understand what generates peak levels of sensed auxin and of MP expression in the leaf. One possibility is that those peaks are the result of the polar, cell-to-cell transport of auxin, which seems to converge on positions of peak MP expression [20, 53, 80–85]. Consistent with this possibility, abnormal positions of *MP* expression domains in developing auxin-transport-inhibited leaves foreshadow the abnormal positions of veins in mature auxin-transport-inhibited leaves [59, 78, 82, 86]. One other possibility is that peak levels of MP expression arise from *MP*’s self-activation — as proposed to happen during embryogenesis [47] and flower formation [87] — and the levels of *MP* expression we measured in MP::MP are consistent with this possibility. Yet another possibility is that — as proposed to happen during xylem differentiation in the leaf [25] or as the *ATHB8*-related *PHABULOSA* does in the root [88] — *ATHB8* controls *MP* expression, such that interpretation of positional information feed back on generation of that information, as it often happens in animals (reviewed in [89]). Broader expression domains of an *MP* expression reporter in *athb8* leaves [16, 90] are consistent with such a possibility. All these possibilities will have to be considered in future work to test whether the gene regulatory network our results suggest is required for restriction of *ATHB8* preprocambial expression to narrow stripes is also sufficient for it.

Finally, it will be interesting to understand whether the incoherent feedforward loop we propose restricts activation of *ATHB8* preprocambial expression to narrow stripes also controls the striped expression of *ATHB8* in other organs and the striped expression of other genes in plants.

## Conclusions

Our results suggest a mechanism by which in plants a broadly expressed transcription factor — MP — activates expression of a target gene — *ATHB8* — in narrow stripes. The very same regulatory mechanism that controls activation of *ATHB8* preprocambial expression in single files of cells is most frequently used in animals to generate stripes of gene expression [91], suggesting unexpected conservation of regulatory logic of striped gene expression in plants and animals despite the independent evolution of their multicellularity. Nevertheless, in animals, such regulatory logic typically leads to activation of target gene expression in a stripe that is outside the expression domain of the activating transcription factor (e.g., [74–76, 92]), whereas *ATHB8* expression is activated in a stripe that is a subset of the MP expression domain. It will be interesting to understand whether these are plant- and animal-specific outputs of the same conserved regulatory logic.

## Methods

### Plants

Origin and nature of lines, genotyping strategies, and oligonucleotide sequences are in Additional File 2: Table S1, Additional File 2: Table S2, and Additional File 2: Table S3, respectively. Seeds were sterilized and sowed as in [93]. Stratified seeds were germinated and seedlings were grown at 22 °C under continuous light (~90 μmol m^−2^ s^−1^). To induce MPΔPB1:GR translocation to the nucleus, seeds were sown on, or 3.75-DAG seedlings were transferred to, dex-supplemented medium (30 μM final concentration). Plants were grown at 25 °C under fluorescent light (~100 μmol m^−2^ s^−1^) in a 16-h-light/8-h-dark cycle and transformed as in [93]. For each construct generated in this study (see Additional File 2: Table S1), the progeny of at least 10 independent transgenic lines were inspected to identify the most representative leaf expression pattern or vein network phenotype. Detailed analysis was performed on the progeny of two homozygous lines per construct. Such representative lines were selected because of strong expression or phenotype emblematic of the profile observed across the entire transgenic series and resulting from single transgene insertion. The same ATHB8::nYFP line (generated in WT background) [16] was introduced in all genetic backgrounds by crossing.

### RT-qPCR

Total RNA was extracted with Qiagen’s RNeasy Plant Mini Kit from 4-day-old seedlings grown in half-strength Murashige and Skoog salts, 15 g l^−1^ sucrose, 0.5 g l^−1^ MES, pH 5.7, at 23 °C under continuous light (~80 μmol m^−2^ s^−1^) on a rotary shaker at 50 rpm. DNA was removed with Invitrogen’s TURBO DNA-free kit, and RNA was stabilized by the addition of 20 U of Thermo Fisher Scientific’s Superase-In RNase Inhibitor. First-strand cDNA was synthesized from ~100 ng of DNase-treated RNA with Thermo Fisher Scientific’s RevertAid Reverse Transcriptase according to the manufacturer’s instructions, except that 50 pmol of Thermo Fisher Scientific’s Oligo(dT)_18_ Primer, 50 pmol of Thermo Fisher Scientific’s Random Hexamer Primer, and 20 U of Superase-In RNase Inhibitor were used. qPCR was performed with Applied Biosystems’ 7500 Fast Real-Time PCR System on 2 μl of 1:3-diluted cDNA with 5 pmol of each gene-specific primers (Additional File 2: Table S3), 2.5 pmol of gene-specific probe (Additional File 2: Table S3), and Applied Biosystems’ TaqMan 2✕ Universal PCR Master Mix in a 10-μl reaction volume. Probe and primers were designed with Applied Biosystems’ Primer Express. Relative *MP* transcript levels were calculated with the 2^−ΔΔCt^ method [94] using *ACTIN2* transcript levels for normalization.

### Imaging

For confocal laser scanning microscopy, developing leaves were mounted and imaged as in [95], except that emission was collected from ~1.5–5.0-μm-thick optical slices. In single-fluorophore marker lines, YFP was excited with the 514-nm line of a 30-mW Ar laser, and emission was collected with a BP 520–555 filter. In multiple-fluorophore marker lines, CFP, QFP, and autofluorescent compounds were excited with the 458-nm line of a 30-mW Ar laser, YFP was excited with the 514-nm line of a 30-mW Ar laser, and RFP was excited with the 543-nm line of a HeNe laser. CFP and QFP emission were collected with a BP 475–525 filter, YFP emission was collected with a BP 520–555 filter, RFP emission was collected between 581 and 657 nm, and autofluorescence was collected between 604 and 700 nm. Signal intensity levels of 8-bit grayscale images acquired at identical settings were quantified in the Fiji distribution of ImageJ [96–99]. To visualize RFP/YFP ratios, the histogram of the YFP images was linearly stretched in the Fiji distribution of ImageJ such that the maximum gray value of the YFP images matched that of the corresponding RFP images, and the RFP images were divided by the corresponding YFP images. GUS activity in developing leaves was detected as in [13]. Stained leaves were fixed, cleared, and mounted as in [13], and mounted leaves were imaged with a Zeiss AxioImager.M1 microscope equipped with a QImaging MicroPublisher 5.0 RTV camera. Mature leaves were fixed, cleared, and mounted as in [54, 78], and mounted leaves were imaged as in [33]. Image brightness and contrast were adjusted by linear stretching of the histogram in in the Fiji distribution of ImageJ.

### Vein network analysis

The cardinality, continuity, and connectivity indices were calculated as in [20]. Briefly, the number of “touch points” (TPs, where a TP is the point where a vein end contacts another vein or a vein fragment), “end points” (EPs, where an EP is the point where an “open” vein — a vein that contacts another vein only at one end — terminates free of contact with another vein or a vein fragment), “break points” (KPs, where a KP is each of the two points where a vein fragment terminates free of contact with veins or other vein fragments), and “exit points” (XPs, where an XP is the point where a vein exits leaf blade and enters leaf petiole) in dark-field images of cleared mature leaves was calculated with the Cell Counter plugin in the Fiji distribution of ImageJ. Because a vein network can be understood as an undirected graph in which TPs, EPs, KPs, and XPs are vertices, and veins and vein fragments are edges, and because each vein is incident to two TPs, a TP and an XP, a TP and an EP, or an XP and an EP, the cardinality index — a measure of the size (i.e., the number of edges) of a graph — is a proxy for the number of veins and is calculated as [(TPs + XPs − EPs)/2] + EPs, or (TPs + XPs + EPs)/2. The continuity index quantifies how close a vein network is to a network with the same number of veins, but in which at least one end of each vein fragment contacts a vein and is therefore calculated as the ratio of the cardinality index of the first network to the cardinality index of the second network: [(TP + XP + EP)/2]/[(TP + XP + EP + KP)/2], or (TP + XP + EP)/(TP + XP + EP + KP). The connectivity index quantifies how close a vein network is to a network with the same number of veins, but in which both ends of each vein or vein fragment contact other veins, and is therefore calculated as the ratio of the number of “closed” veins — those veins which contact vein fragments or other veins at both ends — in the first network to the number of closed veins in the second network (i.e., the cardinality index of the second network): [(TP + XP − EP)/2]/[(TP + XP + EP + KP)/2], or (TP + XP − EP)/(TP + XP + EP + KP). Finally, because the number of meshes in a vein network equals the number of closed veins, the cyclicity index — a proxy for the number of meshes in a vein network — is calculated as: (TP + XP − EP)/2.

## Supplementary Information

**Additional File 1: Figure S1.***ATHB8*-, *SHR*-, and *MP*-Promoter-Driven Expression. (A–F) First leaves 4 DAG. Confocal laser scanning microscopy. Dashed white line: leaf outline. Top right: genotype. Bottom left: reproducibility index (number of samples with the displayed features / number of analyzed samples). (C) Co-expression of ATHB8::nCFP and SHR::nYFP during second loop formation. (E,F) Look-up table — ramp in G — visualizes YFP expression levels; blue: autofluorescence. Scale bars: (A,B,D–F) 25 μm; (C) 10 μm. **Figure S2.** MP::MP:YFP and MP::MP Functionalities in Vein Network Formation. Dark-field illumination of cleared first leaves 14 DAG. Top right: genotype. Scale bars: 0.5 mm. **Figure S3.** *ATHB8* Expression Domains and MP and *RIBO* Expression Levels. First leaves 4 DAG. Confocal laser scanning microscopy. Top right: reporter. Dashed green outline: second loop nuclei expressing ATHB8::nCFP (A,B) or ATHB8::nYFP (D,E). (B,E) Look-up table — ramp in C — visualizes expression levels. Scale bars (shown, for simplicity, only in A and D): 5 μm. **Figure S4.** *ATHB8* Expression Domains and *RIBO* Expression Levels. (A–E) First leaves 4 DAG. (A) Schematic of 4-DAG leaf — imaged in B–E — illustrating onset of *ATHB8* expression (red) — imaged in B — associated with second loop formation [16, 22, 44]. Increasingly darker gray: progressively older *ATHB8* expression domains. (B–E) Confocal laser scanning microscopy. (B) ATHB8::nYFP expression. (C) RIBO::nCFP expression. (D) Autofluorescence. (E) Overlay of images in B–D; red: ATHB8::nYFP expression; green: RIBO::nCFP expression; blue: autofluorescence. (F) RIBO::nCFP expression levels (mean ± SE) in nuclei at positions -2, -1, +1, and +2 — as defined in legend to Fig. 2 — relative to RIBO::nCFP expression levels in nuclei at position 0 — as defined in legend to Fig. 2 — during second loop formation. Difference between RIBO::nCFP expression levels in nuclei at position -2 or -1 and RIBO::nCFP expression levels in nuclei at position 0 was significant at *P* < 0.001 (***) by One-Way ANOVA and Tukey’s Pairwise test. Sample population sizes: 27 leaves; position -2, 42 nuclei; position -1, 64 nuclei; position 0, 69 nuclei; position +1, 50 nuclei; position +2, 28 nuclei. Scale bars (shown, for simplicity, only in column 2): 5 μm. **Figure S5.** *mp-11* and MP::MP Effects on *MP* Expression. *MP* transcript levels in *mp-11* and MP::MP seedlings relative to *MP* transcript levels in WT (mean ± SE of three technical replicates for each of three biological replicates). Seedlings 4 DAG. RT-qPCR. Difference between *mp-11* and WT, and between MP::MP and WT was significant at *P* < 0.001 (***) by *F*-test and *t*-test with Bonferroni correction. **Figure S6.** *ATHB8* Expression Domains and Levels in *iaa12-1* and MP::VP16:bdlΔI;*iaa12-1.* (A,B) First leaves 4 DAG. Confocal laser scanning microscopy. Dashed white line: leaf outline. ATHB8::nYFP expression (look-up table — ramp in C — visualizes expression levels). Top right: genotype. Bottom left: reproducibility index (number of samples with the displayed features / number of analyzed samples). Scale bars: (A,B) 25 μm. **Figure S7.** Summary and Interpretation. A three-gene incoherent type-I feedforward loop [77] activates *ATHB8* expression in narrow preprocambial stripes and leads to vein formation. *MP* receives the auxin input and activates expression of intermediate-loop *AUX*/*IAA* genes, which in turn inhibit *MP* expression [60, 64]. *MP* and *AUX*/*IAA* genes jointly regulate expression of the stripe gene *ATHB8*, which converts the auxin input into vein formation output. Arrows indicate positive effects. Blunt-ended lines indicate negative effects.

**Additional File 2: Table S1.** Origin and Nature of Lines. **Table S2.** Genotyping Strategies. **Table S3.** Oligonucleotide Sequences.

## Data Availability

All data generated or analyzed during this study are included in this published article and its supplementary information files, or are available from the corresponding author on reasonable request.

## Abbreviations

ATHB: ARABIDOPSIS THALIANA HOMEOBOX
AUX/IAA: AUXIN/INDOLE-3-ACETIC-ACID-INDUCIBLE
BDL: BODENLOS
CFP: Cyan fluorescent protein
DAG: Days after germination
dex: Dexamethasone
EAR: Ethylene-responsive-element-binding-protein-associated amphiphilic repression
GFP: Green fluorescent protein
GR: Glucocorticoid receptor
GUS: β-glucuronidase
HD-ZIP III: HOMEODOMAIN-LEUCINE ZIPPER III
miR165a: microRNA165a
MP: MONOPTEROS
PB1: PHOX/BEM1
QFP: Turquoise fluorescent protein
RFP: Red fluorescent protein
RPS5A: RIBOSOMAL PROTEIN S5A
SHR: SHORT-ROOT
VP16: Virus protein 16
WT: Wild type
YFP: Yellow fluorescent protein
