# Supplementary material for: Control of vein-forming, striped gene expression by auxin signaling
Source: BMC Biol. 2021 Sep 24;19:213. doi: 10.1186/s12915-021-01143-9 (PMC8461865; doi:10.1186/s12915-021-01143-9)

ADDITIONAL FILE 1. SUPPLEMENTAL FIGURES S1–S7 AND LEGENDS

Supplemental Figure Legends

Figure S1. ATHB8-, SHR-, *and* MP-*Promoter-Driven Expression*

(A–F) First leaves 4 DAG. Confocal laser scanning microscopy. Dashed white line: leaf outline. Top right: genotype. Bottom left: reproducibility index (number of samples with the displayed features / number of analyzed samples). (C) Co-expression of ATHB8::nCFP and SHR::nYFP during second loop formation. (E,F) Look-up table — ramp in G — visualizes YFP expression levels; blue: autofluorescence. Scale bars: (A,B,D–F) 25 µm; (C) 10 µm.

Figure S2. MP::MP:YFP and MP::MP Functionalities in Vein Network Formation

Dark-field illumination of cleared first leaves 14 DAG. Top right: genotype. Scale bars: 0.5 mm.

Figure S3. ATHB8 *Expression Domains and MP and* RIBO *Expression Levels*

First leaves 4 DAG. Confocal laser scanning microscopy. Top right: reporter. Dashed green outline: second loop nuclei expressing ATHB8::nCFP (A,B) or ATHB8::nYFP (D,E). (B,E) Look-up table — ramp in C — visualizes expression levels. Scale bars (shown, for simplicity, only in A and D): 5 µm.

Figure S4. ATHB8 *Expression Domains and* RIBO *Expression Levels*

(A–E) First leaves 4 DAG. (A) Schematic of 4-DAG leaf — imaged in B–E — illustrating onset of *ATHB8* expression (red) — imaged in B — associated with second loop formation (16, 22, 44). Increasingly darker gray: progressively older *ATHB8* expression domains. (B–E) Confocal laser scanning microscopy. (B) ATHB8::nYFP expression. (C) RIBO::nCFP expression. (D) Autofluorescence. (E) Overlay of images in B–D; red: ATHB8::nYFP expression; green: RIBO::nCFP expression; blue: autofluorescence. (F) RIBO::nCFP expression levels (mean ± SE) in nuclei at positions -2, -1, +1, and +2 — as defined in legend to Figure 2 — relative to RIBO::nCFP expression levels in nuclei at position 0 — as defined in legend to Figure 2 — during second loop formation. Difference between RIBO::nCFP expression levels in nuclei at position -2 or -1 and RIBO::nCFP expression levels in nuclei at position 0 was significant at $P<0.001$ (***) by One-Way ANOVA and Tukey's Pairwise test. Sample population sizes: 27 leaves; position -2, 42 nuclei; position -1, 64 nuclei; position 0, 69 nuclei; position 1, 50 nuclei; position 2, 28 nuclei. Scale bars (shown, for simplicity, only in column 2): 5 μm.

Figure S5. mp-11 *and MP::MP Effects on* MP *Expression*

MP transcript levels in *mp-11* and MP::MP seedlings relative to *MP* transcript levels in WT (mean ± SE of three technical replicates for each of three biological replicates). Seedlings 4 DAG. RT-qPCR. Difference between *mp-11* and WT, and between MP::MP and WT was significant at $P<0.001$ (***) by F-test and t-test with Bonferroni correction.

Figure S6. ATHB8 *Expression Domains and Levels in* iaa12-1 *and MP::VP16:bdlΔI;*iaa12-1

(A,B) First leaves 4 DAG. Confocal laser scanning microscopy. Dashed white line: leaf outline. ATHB8::nYFP expression (look-up table — ramp

in C — visualizes expression levels). Top right: genotype. Bottom left: reproducibility index (number of samples with the displayed features / number of analyzed samples). Scale bars: (A,B) 25 μm.

Figure S7. Summary and Interpretation.

A three-gene incoherent type-I feedforward loop (77) activates *ATHB8* expression in narrow preprocambial stripes and leads to vein formation. *MP* receives the auxin input and activates expression of intermediate-loop *AUX/IAA* genes, which in turn inhibit *MP* expression (60, 64). *MP* and *AUX/IAA* genes jointly regulate expression of the stripe gene *ATHB8*, which converts the auxin input into vein formation output. Arrows indicate positive effects. Blunt-ended lines indicate negative effects.

Supplemental Figures

Figure S1

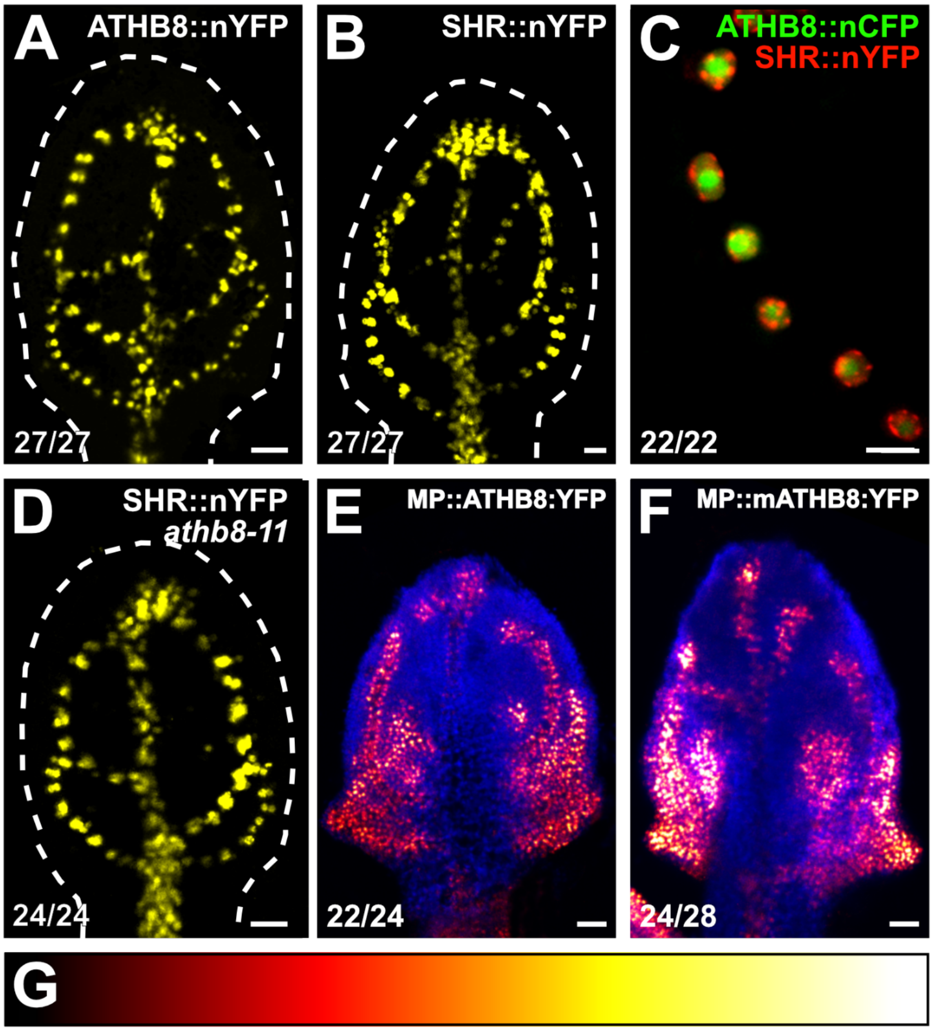

Figure S2

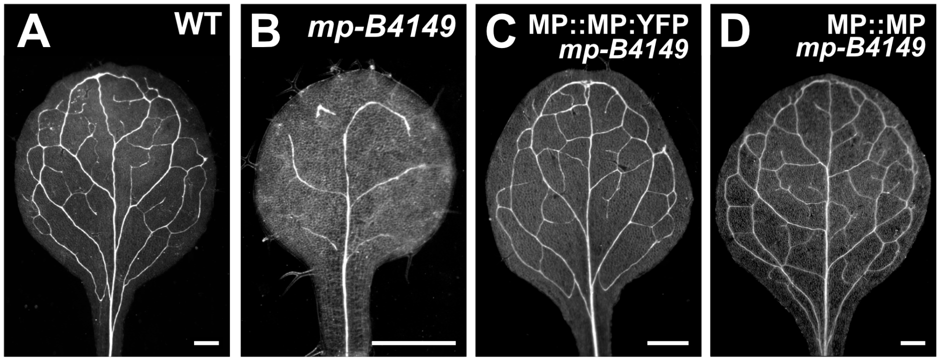

Figure S3

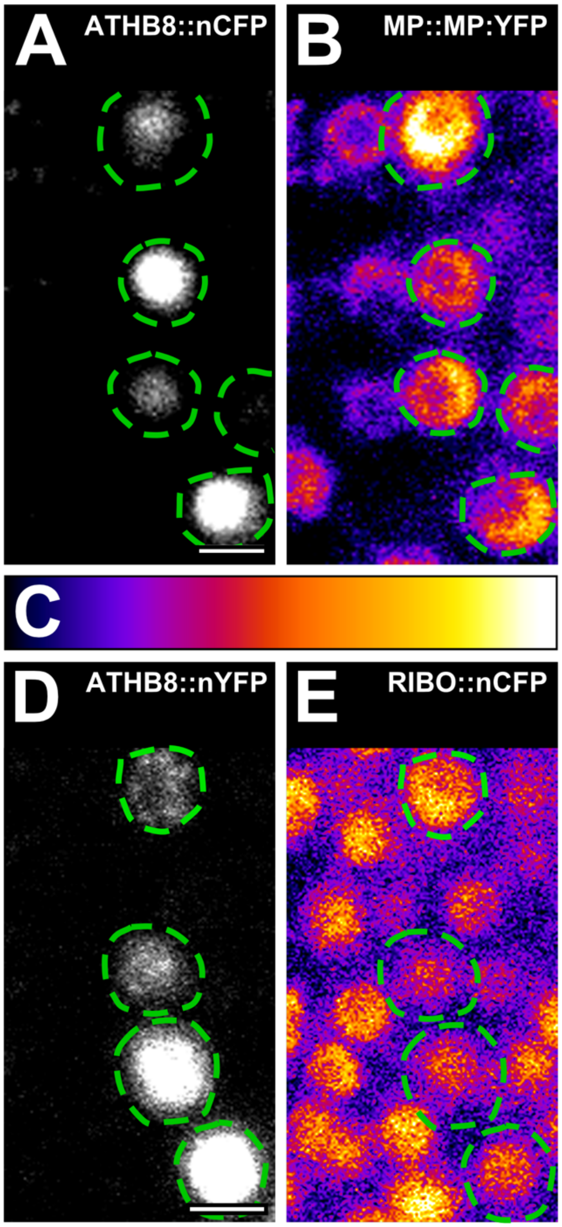

Figure S4

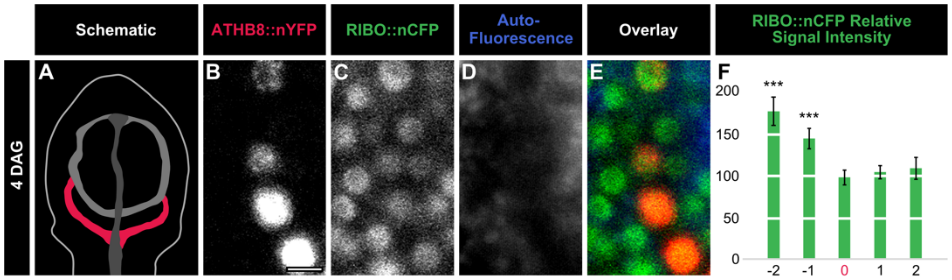

Schematic
ATHB8::nYFP
RIBO::nCFP
Auto-Fluorescence
Overlay
RIBO::nCFP Relative Signal Intensity
4 DAG
A
B
C
D
E
F
200
150
100
50
0
***
***
-2
-1
0
1
2

Figure S5

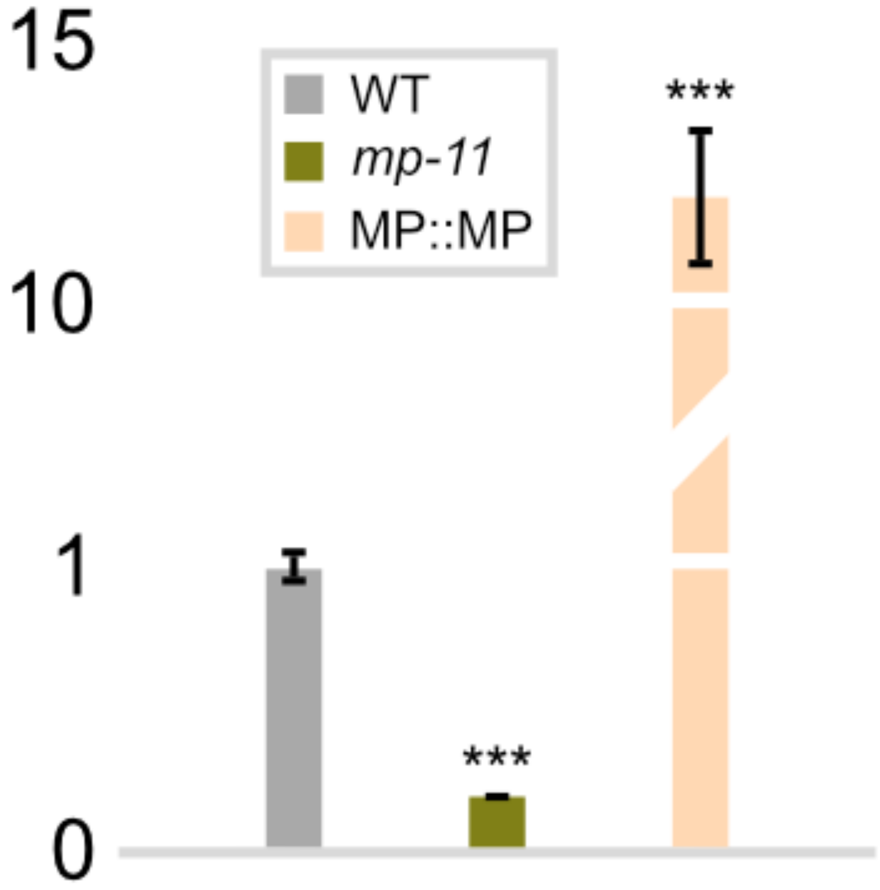

Figure S6

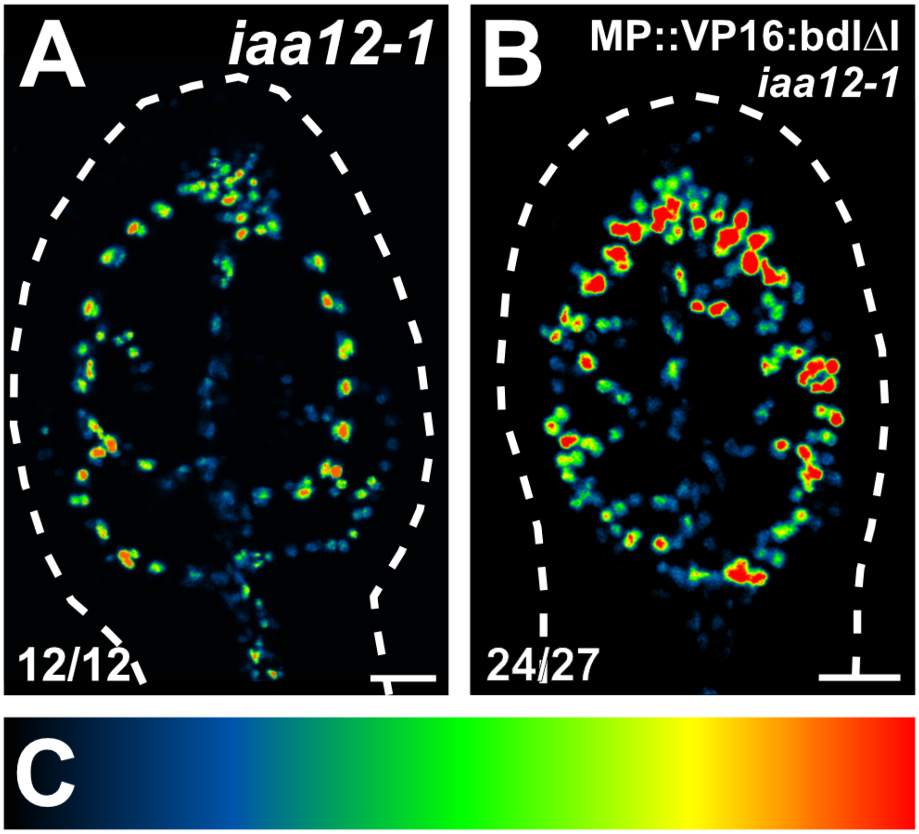

Figure S7

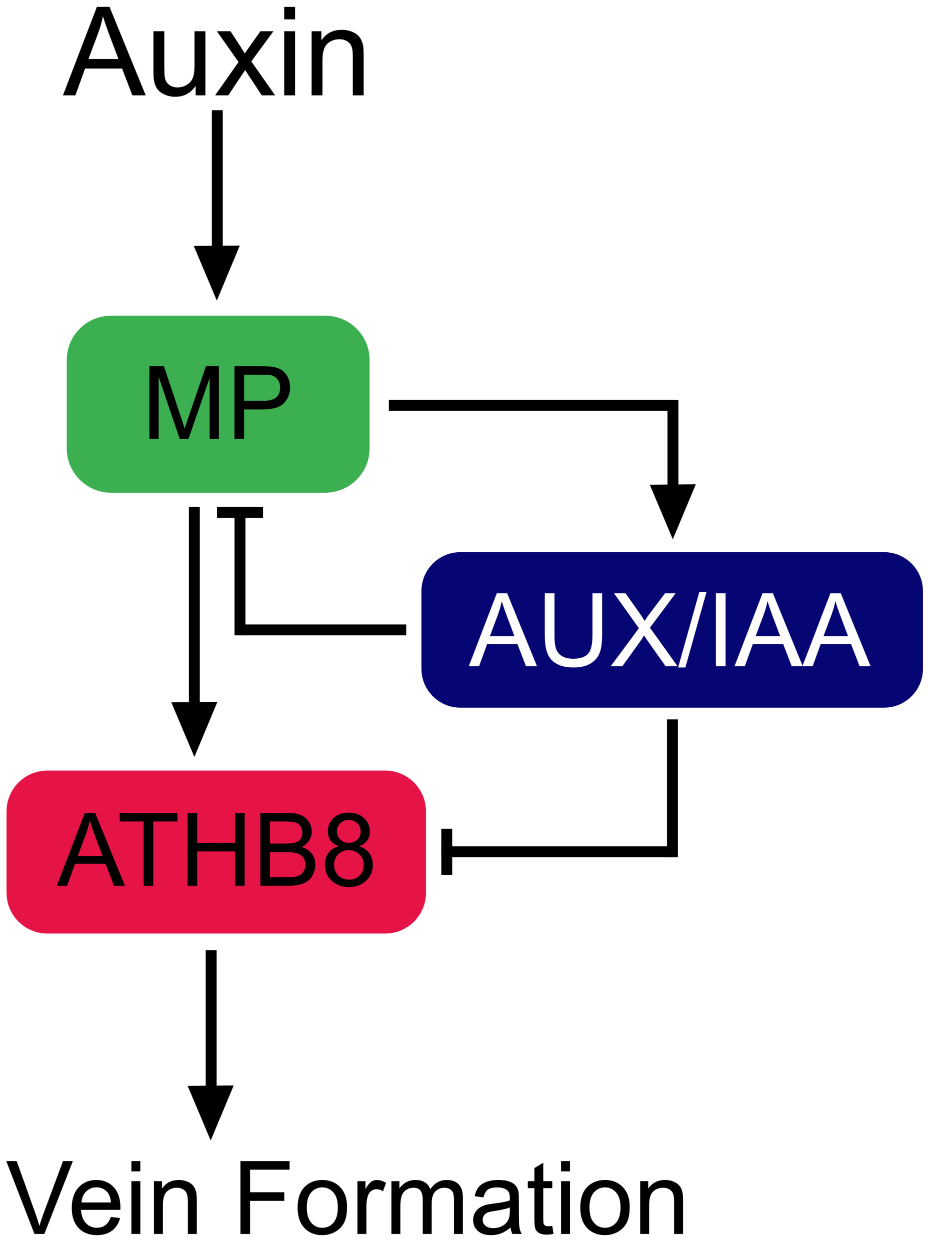

Supplement: Supplementary file 1 — Additional File 1: Figure S1.ATHB8-, SHR-, and MP-Promoter-Driven Expression. (A–F) First leaves 4 DAG. Confocal laser scanning microscopy. Dashed white line: leaf outline. Top right: genotype. Bottom left: reproducibility index (number of samples with the displayed features / number of analyzed samples). (C) Co-expression of ATHB8::nCFP and SHR::nYFP during second loop formation. (E,F) Look-up table — ramp in G — visualizes YFP expression levels; blue: autofluorescence. Scale bars: (A,B,D–F) 25 μm; (C) 10 μm. Figure S2. MP::MP:YFP and MP::MP Functionalities in Vein Network Formation. Dark-field illumination of cleared first leaves 14 DAG. Top right: genotype. Scale bars: 0.5 mm. Figure S3. ATHB8 Expression Domains and MP and RIBO Expression Levels. First leaves 4 DAG. Confocal laser scanning microscopy. Top right: reporter. Dashed green outline: second loop nuclei expressing ATHB8::nCFP (A,B) or ATHB8::nYFP (D,E). (B,E) Look-up table — ramp in C — visualizes expression levels. Scale bars (shown, for simplicity, only in A and D): 5 μm. Figure S4. ATHB8 Expression Domains and RIBO Expression Levels. (A–E) First leaves 4 DAG. (A) Schematic of 4-DAG leaf — imaged in B–E — illustrating onset of ATHB8 expression (red) — imaged in B — associated with second loop formation [16, 22, 44]. Increasingly darker gray: progressively older ATHB8 expression domains. (B–E) Confocal laser scanning microscopy. (B) ATHB8::nYFP expression. (C) RIBO::nCFP expression. (D) Autofluorescence. (E) Overlay of images in B–D; red: ATHB8::nYFP expression; green: RIBO::nCFP expression; blue: autofluorescence. (F) RIBO::nCFP expression levels (mean ± SE) in nuclei at positions -2, -1, +1, and +2 — as defined in legend to Fig. 2 — relative to RIBO::nCFP expression levels in nuclei at position 0 — as defined in legend to Fig. 2 — during second loop formation. Difference between RIBO::nCFP expression levels in nuclei at position -2 or -1 and RIBO::nCFP expression levels in nuclei at position 0 was sig [file 12915_2021_1143_MOESM1_ESM.pdf]
