# Supplementary material for: Control of vein-forming, striped gene expression by auxin signaling
Source: BMC Biol. 2021 Sep 24;19:213. doi: 10.1186/s12915-021-01143-9 (PMC8461865; doi:10.1186/s12915-021-01143-9)
Supplement: Supplementary file 2 — Additional File 2: Table S1. Origin and Nature of Lines. Table S2. Genotyping Strategies. Table S3. Oligonucleotide Sequences. [file 12915_2021_1143_MOESM2_ESM.pdf]

ADDITIONAL FILE 2. SUPPLEMENTAL TABLES S1–S3

Table S1. Origin and Nature of Lines

LINE	ORIGIN / NATURE
athb8-11	ABRC (CS6969); (19); WT at the *ER* (AT2G26330) locus
athb8-27	ABRC (CS111153)
SHR::nYFP	(22)
ATHB8::nCFP	(14)
SHR::miR165a	Transcriptional fusion of *SHR* (AT4G37650; -2505 to -10; primers: "SHR HindIII F" and "SHR SalI R") to miR165a (AT1G01183; - 138 to +323 relative to the transcriptional start-site; primers: "SalI FWD – MiRNA 165" and "KpnI REV – MiRNA 165")
SHR::mATHB8	(27)
SHR::mATHB8:EAR	Translational fusion of SHR::mATHB8 (27) (primers: "SalI SHR Promoter FP" and "XhoI mATHB8 RP") to the sequence encoding the EAR portable repressor domain (26) (primers: "EAR XhoI + KpnI Forward" and "EAR Reverse")

MP::ATHB8	Transcriptional fusion of *MP* (AT1G19850; -3281 to -1; primers: "MP BamHI Fwd" and "MP KpnI Rev") to the *ATHB8* (AT4G32880) cDNA (GeneBank accession: BT008798; ABRC: U24724; +1 to +2502; primers: "ATHB8 cDNA KpnI FWD" and "ATHB8 cDNA SmaI Rev")
MP::mATHB8	Transcriptional fusion of *MP* (AT1G19850; -3281 to -1; primers: 63 "MP BamHI Fwd" and "MP KpnI Rev") to the *ATHB8* (AT4G32880) cDNA (GeneBank accession: BT008798; ABRC: U24724; +1 to +2502; primers: "ATHB8 cDNA KpnI FWD" and "ATHB8 cDNA SmaI Rev"; "ATHB8mut165FWD" and "ATHB8mut165REV")
MP::MP:YFP	Translational fusion of *MP* (AT1G19850; -3281 to +3815; primers: "MP Prom SalI Fwd" and "MP KpnI Rev-2"; "MP 3 kb SalI Fwd" and "MP 3 kb XhoI Rev") to the sequence encoding EYFP (primers: "ECFP AflII F" and "ECFP AflII R"); rescues the root (240/240 seedlings), vein (Additional File 1: Figure S1), and inflorescence (160/160 plants) defects of *mp-B4149*
mp-B4149	(30)
RIBO::nCFP	ABRC (CS23898); (31); WT at the *ER* (AT2G26330) locus
ATHB8::nYFP	(14)
mp-U55	ABRC (CS8147); (16, 32)

mp-11	(33)
MP::MP	*MP* (AT1G19850; -3281 to +3830; primers: "MP Prom SalI Fwd" and "MP KpnI Rev-2"; "MP 3KB SalI Fwd" and "MP 3kb XhoI Rev"); rescues the root (169/176 seedlings), vein (Additional File 1: Figure S1), and inflorescence (6/6 plants) defects of *mp-B4149*
bdl	(34); introgressed into Col-0
MP::VP16:bdlΔI	Transcriptional fusion of *MP* (AT1G19850; -3281 to -1; primers: "MP BamHI Fwd" and "MP KpnI Rev-1") to a translational fusion of the sequence encoding the activation domain of the *Herpes simplex* virus protein 16 (VP16) (35) (primers: "VP16 NcoIF2" and "VP16 PstIR") to a 5′-terminally deleted *bdl* (36) (+94 to +1229; primers: "BDL PstIF" and "BDL BamHIR"; "BDL mut F1", "BDL mut F2", "BDL mut F3", "BDL mut F4", "BDL PstIF", and "BDL MfeI mut R"; "BDLd1 PstI F" and "BDL BAMHI R")
iaa12-1	ABRC (CS25213); (37)
tpl-1	ABRC (CS65909); (38)
MP::MPΔPB1:GR	Translational fusion of *MP* (AT1G19850; -3427 to +2388; primers: "MP SalI Forward – Primer # 2" and "MP EcoRI Reverse") to the sequence encoding a fragment of the rat glucocorticoid receptor (GR) (39) (primers: "SpeI GR Forward" and "SacII + KpnI (Internal) GR Reverse")

BDL::nGFP	(39)
BDL::BDL:GFP	(40)
BDL::bdl:GUS	(41)
BDL::bdl:YFP	Translational fusion of *bdl* (AT1G04550; -2431 to +1541; primers: "BamHI BDL Domains 1+2 Forward" and "EcoRI BDL Domains 1+2 Reverse"; "SalI Domains 3+4 Forward" and "KpnI Domains 3+4 Reverse"; "BDL::Venus Site-Directed Mutagenesis 1" and "BDL::Venus Site-Directed Mutagenesis 2") to the sequence encoding VENUS (primers: "EcoRI Venus Forward" and "SalI Venus Reverse")
ATHB8::nQFP	Transcriptional fusion of *ATHB8* (AT4G32880; -2070 to -1; primers: "SalI 2KB ATHB8 Promoter Forward" and "ApaI 2KB ATHB8 Promoter Reverse") to the sequence encoding 2xmTQ2-N7 (primers: "ApaI 2xmTurqoise Forward" and "KpnI 2xmTFP Reverse")
R2D2	(42)
[TGTCTG]::nYFP	(16)
[TAGCTG]::nYFP	(16)
[TGTCAG]::nYFP	Transcriptional fusion of *ATHB8* (AT4G32880; -953 to -1; primers: "1NagARE" and "Athb8 R-5") to the sequence encoding HTA6:EYFP (43)

[TGTCTG]::nYFP	Transcriptional fusion of *ATHB8* (AT4G32880; -953 to -1; primers: "1NcARE" and "Athb8 R-5") to the sequence encoding HTA6:EYFP (43)

Table S2. Genotyping Strategies

LINE	STRATEGY
athb8-11	ATHB8: “Athb8 0.5” and “athb8attB2R”; *athb8-11*: “athb8 -5944” and “PD991- RB
athb8-27	ATHB8: “athb8-27 RP” and “athb8-27 LP”; *athb8-27*: “athb8-27 RP” and “Spm32”
mp-B4149	“MP 1498-s” and “MP2082-AS”; *Mse*I
mp-U55	“MP Seq 2061” and “U55 Geno Rev”; *Sml*I
mp-11	*MP*: “Sail_1265_F06LP” and “Sail_1265_F06RP”; *mp-11*: “LB3” and “Sail_1265_F06RP”
bdl	“bdl geno F” and “bdl geno R”; *Hae*III
iaa12-1	*IAA12*: “SALK_138684 LP” and “SALK_138684 RP”; *iaa12-1*: “LBb1.3” and “SALK_138684 RP”
tpl-1	“tpl Caps Genotyping Forward” and “tpl Caps Genotyping Reverse”; *Nco*I

Table S3. Oligonucleotide Sequences

NAME	SEQUENCE (5' TO 3')
SHR HindIII F	GAGAAGCTTGACAAAGAAGCAGAGCGTGG
SHR SalI R	TGGGTCGACTTAATGAATAAGAAAATGAATAGAAGAAAGGG
SalI FWD – MiRNA 165	ATTGTCGACCCACTCATCATTCCCTCATC
KpnI REV – MiRNA 165	AGCGGTACCCTTATAGAAAATACTTCGTTAGCTTG
SalI SHR Promoter FP	GGGGTCGACACATAAACCAGTAGACAT
XhoI mATHB8 RP	GGGCTCGAGTATAAAAGACCAGTTGAGG
EAR XhoI + KpnI Forward	TCGAGCTAGATCTGGATCTAGAACTCCGTTTGGGTTTCGCTTAAGGTAC
EAR Reverse	CTTAAGCGAAACCCAAACGGAGTTCTAGATCCAGATCATGC
MP BamHI Fwd	AAGGGATCCTCCGGGTTAATCAGTATTATTAC
MP KpnI Rev	ACAGGTACCACAGAGAGATTTTTCAATGTTCTG
ATHB8 cDNA KpnI FWD	GTCGGTACCATGGGAGGAGGAAGCAATAATAG
ATHB8 cDNA SmaI Rev	ATGCCCGGGATCATATAAAAGACCAGTTGAGG
ATHB8mut165FWD	ATAGGAATCGTTGCTATTTCTC

ATHB8mut165REV	GGAATCTGGTCCAGGCTTCATC
MP Prom SalI Fwd	CCCGTCGACGTATATATAAACAATACCACCTTATAAC
MP KpnI Rev-2	CATGGTACCTGCAGAATTAGCATACCACAC
MP 3 kb SalI Fwd	TCTGTCGACTCCGGGTTAATCAGTATTATTAC
MP 3 kb XhoI Rev	ATTCTCGAGTTAAGAGTTAAGACCACCTCC
ECFP AflII F	TTACTTAAGGTGAGCAAGGGCGACGAGC
ECFP AflII R	AGACTTAAGATTGTACAGCTCGTCCATGCC
VP16 NcoIF2	TTACCATGGCCCCCCCGACCGATGTC
VP16 PstIR	TTTCTGCAGCCCCACCGTACTCGTCAATTC
BDL PstIF	ATACTGCAGCTCGTGGTGTGTCAGAATTGGAC
BDL BamHIR	TACGGATCCACTAAACTGGGTTGTTTCTTTGTC
BDL mut F1	AATCTTCCGGCGGAGAGTGTTAGAGAATTGGG
BDL mut F2	GTGGGTAAAAGTAATCTTCCGGCGGAGAGTG
BDL mut F3	GTGTCAGAATTGGAGGTGGGTAAAAGTAATCTTCCG
BDL mut F4	CGTGGTGTGTCAGAATTGGAGGTGGGGAAGAGTAATC
BDL MfeI mut R	TAACAATTGGTGACCATCCTACCACTTGAC
BDLd1 PstI F	AAACTGCAGCGTGGAAAGAGCGTGGG

MP SalI Forward – Primer # 2	GGGGTCGACCGGATTCGTGATCTTCGTATCCCAT
MP EcoRI Reverse	ATTGAATTCGGTTCGGACGCGGGGTGTCGCAATT
SpeI GR Forward	GGGACTAGTGGAGAAGCTCGAAAAACAAAG
SacII + KpnI (Internal) GR Reverse	AATCCGCGGGGTACCTCATTTTTGATGAAACAGAAG
BamHI BDL Domains 1+2 Forward	AAAGGATCCATGTGGTAGTGTCGAGAAGGG
EcoRI BDL Domains 1+2 Reverse	CCTGAATTCGAGCTCATCATTCTTCACAAC
SalI Domains 3+4 Forward	AGCGTCGACAAAGATGTGTCAATGAAGGTGAAT
KpnI Domains 3+4 Reverse	GCTGGTACCCTTTCTTTGGAATCATAAACATAACTC
BDL::Venus Site-Directed Mutagenesis 1	ACTTGACTGCCACAACAAACCAAGA
BDL::Venus Site-Directed Mutagenesis 2	GGTAGGATGGTCACCAATTGGGTTACA
EcoRI Venus Forward	GCGGAATTCGTGAGCAAGGGCGAGGAG
SalI Venus Reverse	GATGTCGACCTTGTACAGCTCGTCCATGC
SalI 2KB ATHB8 Promoter Forward	CGCGTCGACCATTATAAATATCACGACTGTA

ApaI 2KB ATHB8 Promoter Reverse	ATTGGGCCCCTTTGATCCTCTCCGATCTCT
ApaI 2xmTurqoise Forward	ATTGGGCCCATGGTGAGCAAGGGCGAGGA
KpnI 2xmTFP Reverse	CGAGGTACCTCACTCTTCTTCTTGATCAGCTTCTG
1NagARE	GGGGACAAGTTTGTACAAAAAAGCAGGCTTGGTTGT CTCGTATTAAGGG
Athb8 R-5	GGGGACCACTTTGTACAAGAAAGCTGGGTCTTTGAT CCTCTCCGATCTCTC
1NcARE	GGGGACAAGTTTGTACAAAAAAGCAGGCTTGGTTAC CTGGTATTAAGGG
athb8-27 FP	TGTGAAGAATGGATCCACCTC
athb8-27 RP	AGTGGTCAACACCACTTGACC
Spm32	TACGAATAAGAGCGTCCATTTTAGAGTG
Athb8 0.5	GGGGACAAGTTTGTACAAAAAAGCAGGCTTCCTTTG CTTCCAGAGACCAGCG
athb8attB2R	GGGGACCACTTTGTACAAGAAAGCTGGGTCTTTGAT CCTCTCCGATCTCTC
athb8 -5944	GGTTTGGCATAAAAGTGCGG
PD991- RB	AAAACCTGGCGTTACCCAACT
MP 1498-s	CTCTCAGCGGATAGTATGCACATCGG

MP2082-AS	ATGGATGGAGCTGACGTTTGAGTTC
MP Seq 2061	CATAATGTTACTCTTCATGTACGCC
U55 Geno Rev	GTGCTGTTTGTTGGCGATTGG
Sail_1265_F06LP	GCTTCATCTCTTCAAGCAAGG
Sail_1265_F06RP	TCCCAAAGTCTCACCACTCAC
LB3	TAGCATCTGAATTTCATAACCAATCTCGATACAC
bdl geno F	GCTCAAATCTTGTGATGTGAGTG
bdl geno R	AGTCCACTAGCTTCTGAGGTTCCC
SALK_138684 LP	GTGGGGAAGAGTAATCTTCCG
SALK_138684 RP	CTTCTGCTCTTGACGTCTTGG
LBb1.3	ATTTTGCCGATTTCGGAAC
tpl Caps Genotyping Forward	GCCCTGAAAATGACATCGGT
MP PrimeTime Probe	/56-FAM/CAGACTCAC/ZEN/ AGGCCTTCTCTCGCCA/3IABKFQ/
MP PrimeTime Primer 2	TGTACCAGTGCCTCCAGAATTATC
MP PrimeTime Primer 1	TCCAGTCGCAGATCACATCAG
ACT2 PrimeTime Probe	/56-FAM/ACAGCACTT/ZEN/ GCCCAAGAGCATGA/3IABKFQ/

ACT2 PrimeTime Primer 2	TACTTCCTTTCAGGTGGTGCA
ACT2 PrimeTime Primer 1	GCTGACCGTATGAGCAAAGAAAT
